# Cranial shape evolution of extant and fossil crocodile newts and its relation to reproduction and ecology

**DOI:** 10.1111/joa.13201

**Published:** 2020-04-15

**Authors:** Peter Pogoda, Marcus Zuber, Tilo Baumbach, Rainer R. Schoch, Alexander Kupfer

**Affiliations:** ^1^ Department of Zoology State Museum of Natural History Stuttgart Stuttgart Germany; ^2^ Comparative Zoology Institute of Evolution and Ecology Eberhard Karls University Tuebingen Tuebingen Germany; ^3^ Institute for Photon Science and Synchrotron Radiation (IPS) Karlsruhe Institute of Technology (KIT) Eggenstein‐Leopoldshafen Germany; ^4^ Laboratory for Applications of Synchrotron Radiation (LAS) Karlsruhe Institute of Technology (KIT) Karlsruhe Germany; ^5^ Department of Paleontology State Museum of Natural History Stuttgart Stuttgart Germany; ^6^ Institute of Zoology University of Hohenheim Stuttgart Germany

**Keywords:** *Chelotriton*, cranium, *Echinotriton*, fossil salamander, geometric morphometrics, paleoecology, reproductive biology, shape evolution, *Tylototriton*

## Abstract

The diversity of the vertebrate cranial shape of phylogenetically related taxa allows conclusions on ecology and life history. As pleurodeline newts (the genera *Echinotriton*, *Pleurodeles* and *Tylototriton*) have polymorphic reproductive modes, they are highly suitable for following cranial shape evolution in relation to reproduction and environment. We investigated interspecific differences externally and differences in the cranial shape of pleurodeline newts via two‐dimensional geometric morphometrics. Our analyses also included the closely related but extinct genus *Chelotriton* to better follow the evolutionary history of cranial shape. *Pleurodeles* was morphologically distinct in relation to other phylogenetically basal salamanders. The subgenera within *Tylototriton* (*Tylototriton* and *Yaotriton*) were well separated in morphospace, whereas *Echinotriton* resembled the subgenus *Yaotriton* more than *Tylototriton*. Oviposition site choice correlated with phylogeny and morphology. Only the mating mode, with a random distribution along the phylogenetic tree, separated crocodile newts into two morphologically distinct groups. Extinct *Chelotriton* likely represented several species and were morphologically and ecologically more similar to *Echinotriton* and *Yaotriton* than to *Tylototriton* subgenera. Our data also provide the first comprehensive morphological support for the molecular phylogeny of pleurodeline newts.

## Graphical abstract

We applied geometric morphometrics to cranial morphology in extant and extinct crocodile newts. Our data provides the first comprehensive morphological support for the molecular phylogeny of these newts. Further, we could show that life history traits correlate with head shape and that fossil Chelotriton show similarities in shape with some extant taxa allowing conclusions on its ecology.

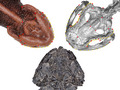

## 1 INTRODUCTION

One of the most complex structures in tetrapods is the cranium, an almost static component. It is composed of various different bones that fuse during ontogeny to build a robust capsule for most of the sensory organs and functional units for various tasks such as food intake and the perception of the environment. This variety of tasks and the structural design forced and allowed vertebrates to evolve very distinct cranial morphologies. Accordingly, the analogous evolution of similar shapes in phylogenetically non‐related taxa can be used to draw conclusions on their ecology, especially the niches they occupy (e.g. Herrel et al., 2004; Dean et al., 2007; Herrel et al., 2008). Despite its enormous morphological variation, the cranial skeleton still allows conclusions on a species´ ecology, e.g. the dentition is usually closely related to the diet (Hotton, 1955; Strait, 1993), and this relation is of special importance for the reconstruction of ancient lineages. As the skeleton usually represents the only remains in the fossil record, it is most promising to carry out comparative osteology that includes extant taxa to draw conclusions about the ecology of extinct vertebrates. Furthermore, by linking morphological traits of extant species to their environment, ecology and life history in a comparative way, it is possible to obtain insights into the evolutionary history of extinct taxa. For example, patterns of countershading in extant species allow conclusions to be made concerning predator–prey interactions or the habitat of dinosaurs (Brown et al., 2017; Smithwick et al., 2017).

True salamanders of the family Salamandridae evolved a variety of cranial shapes (Ivanović and Arntzen, 2017). The taxon of Pleurodelini, often referred to as ‘primitive newts’, represent a basal group of Salamandridae comprising three extant genera (Zhang et al., 2008; Veith et al., 2018). Whereas the three species of ribbed newts, genus *Pleurodeles* Michahelles, 1830, inhabit a restricted range in southwest Europe and North Africa along the Mediterranean, crocodile newts are much more diverse and are currently assigned to two genera. *Echinotriton* Nussbaum and Brodie, 1982 comprises three species, inhabiting the Ryu‐Kyu archipelago, Japan and east China (Chang, 1932; Hou et al., 2014). *Tylototriton* Anderson, 1871 includes 25 species divided into two subgenera: *Tylototriton* Anderson, 1871 and *Yaotriton* Dubois and Raffaëlli, 2009 (Dubois and Raffaelli, 2009). *Tylototriton* is widely distributed from east Nepal to east and central China, southwards to Myanmar, central Vietnam, Laos and Thailand (Wang et al., 2018; Zaw et al., 2019). Apparently, crocodile newts have a quite conservative morphological evolution (Hernandez et al., 2018), leading to a high number of species mainly recognized by genetic studies in recent years. On the other hand, distinct diagnostic morphological characters are only sparsely available (e.g. Nishikawa et al., 2013; Phimmachak et al., 2015a; Qian et al., 2017; Grismer et al., 2018; Zaw et al., 2019).

**FIGURE 1:**
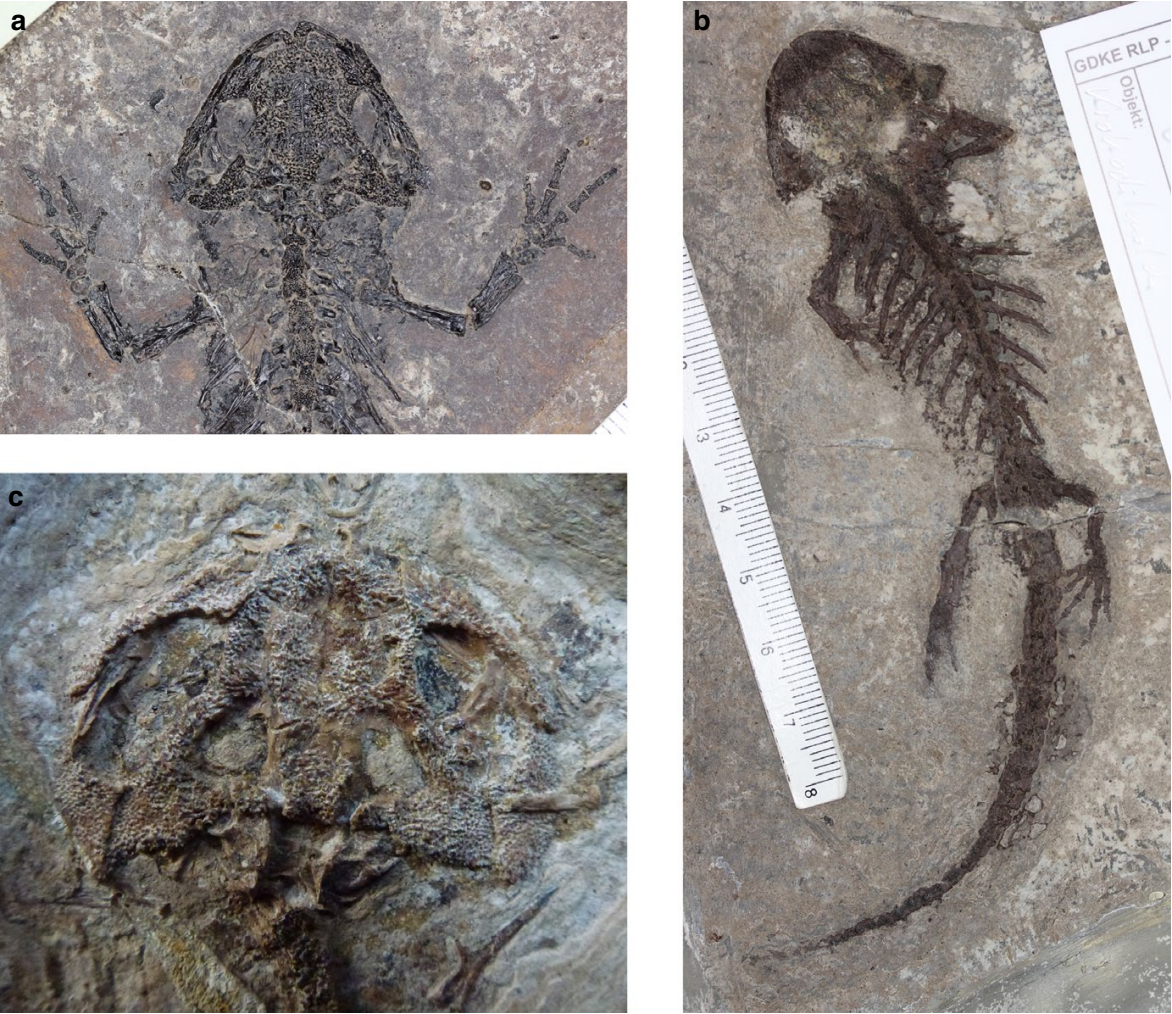
Well‐preserved cranial skeletons of fossil *Chelotriton* specimens from the Enspel crater lake, Rhineland‐Palatine (a,b) and the deposits of the Randeck Maar, Baden‐Wuerttemberg (c), Germany

Pleurodeline newts are polymorphic in their reproductive mode and mating strategy (Kieren et al., 2018), including terrestrial and aquatic mating, as well as the choice of oviposition sites (Kuzmin et al., 1994; Ziegler et al., 2008; Igawa et al., 2013; Bernardes et al., 2017). Whereas some species use a ventral amplexus similar to terrestrial salamandridae, others perform a circular mating dance comparable to European newts (Dasgupta, 1994; Roy and Mushahidunnabi, 2001; Fleck, 2010a; 2010b; Wang et al., 2017; Gong et al., 2018). Ribbed and crocodile newts occupy various habitats along the latitudinal and altitudinal gradient from tropical lowland rainforests to montane forests and grassy landscapes (Bernardes et al., 2013; Hernandez et al., 2017; Hernandez et al., 2019). Their diverse ecology may result in indistinct morphological adaptations hard to access with traditional morphological approaches. Additionally, pleurodeline newts are represented by several fossil taxa. Three extinct species of *Tylototriton* were described from Germany (Noble, 1928; Herre, 1935; 1949), today being recognized as members of other fossil newt genera (Estes, 1981; Nussbaum and Brodie, 1982; Böhme and Ilg, 2003). The most prominent one is the genus *Chelotriton* Pomel, 1853, currently consisting of four nominally described species (Goldfuss, 1831; Pomel, 1853; Westphal, 1980; Bailon, 1989) of which *Chelotriton* *paradoxus* is the best known. *Chelotriton* is known from Spain to east Europe from the Eocene to Miocene (about 50–11 mya). Based on unique morphological characters, *Chelotriton* was assigned to the tribe Pleurodelini by various authors and is regarded as more closely related to crocodile newts, i.e. the genera *Echinotriton* and *Tylototriton,* than to *Pleurodeles* (Marjanović and Witzmann, 2015; Schoch et al., 2015). In recent years, several exceptionally well‐preserved specimens of *Chelotriton* have been excavated from localities in southwest Germany (Figure 1; Roček and Wuttke, 2010; Schoch et al., 2015).

Via two‐dimensional (2D) geometric morphometrics of external head and skull morphology, accessed via micro‐computed tomography (µCT) scans, we investigated how cranial shape of ribbed and crocodile newts differs interspecifically. We tested how cranial morphology relates to selected ecological and reproductive traits. Fossil *Chelotriton* specimens from deposits of Randeck Maar and Enspel Crater Lake were included in the analyses of cranial morphology to obtain further hints on the relationship between extant and extinct taxa and to draw conclusions on the ecology of *Chelotriton* based on morphology–ecology correlations of extant taxa. The overall aim was to obtain novel insights into the evolution of cranial shape in relation to ecology of selected, phylogenetically basal salamandrids.

## 2 MATERIALS AND METHODS

We have investigated the crania of 157 newt specimens covering 21 of 31 extant species (68%) which are currently ascribed to the Pleurodelini (Frost, 2018). With additional data from the literature, we covered information on up to 26 species (see below and Table 1). As populations of *Echinotriton andersoni* originating from the island of Okinawa and the Amami archipelago showed deep divergence and are under debate as to whether they should be recognized as distinct taxonomic units (Hayashi et al., 1992; Honda et al., 2012; Kurabayashi et al., 2012), we treated those separately in our analyses. To exclude additional variation due to sexual dimorphism in extant species, only male specimens were analysed, except for a photograph of the *Echinotriton maxiquadratus* holotype, which is a female. Additionally, eight well‐preserved fossil specimens of the genus *Chelotriton* from deposits of the Randeck Maar, Baden‐Wuerttemberg (17–15 Ma, mammal zone MN5, see Böhme, 2003; Rasser et al., 2013) and Enspel Crater lake, Rhineland‐Palatine (24.8–24.6 Ma, mammal zone MP28, see Roček and Wuttke, 2010; Schindler and Wuttke, 2010), both in Germany, were included in the analysis (Figure 1; Schoch et al., 2015). Currently, specimens of *Chelotriton* from these deposits are tentatively associated with the type species *C. paradoxus* (Schoch et al., 2015).

**TABLE 1:** Sample sizes per species of pleurodeline salamandrids for 2D geometric morphometrics analyses of cranial morphology

Species	*n* – external	*n* – osteology
*Echinotriton andersoni* – Okinawa	8	5
*Echinotriton andersoni* – Amami	4	3
***Echinotriton maxiquadratus***	1*	–
***Tylototrition (Tylototriton) anguliceps***	5*	2
***Tylototrition (Yaotriton) asperrimus***	6	7
***Tylototrition (Yaotriton) broadoridgus***	1*	–
*Tylototrition (Yaotriton) hainanensis*	1	1
*Tylototrition (Tylototriton) himalayanus*	12	9
***Tylototrition (Tylototriton) kachinorum***	7*	–
*Tylototrition (Tylototriton) kweichowensis*	10	5
***Tylototrition (Yaotriton) liuyangensis***	1*	–
*Tylototrition (Yaotriton) lizhenchangi*	3	2
***Tylototrition (Tylototriton) ngarsuensis***	2*	–
***Tylototrition (Yaotriton) notialis***	2*	1
*Tylototrition (Yaotriton) panhai*	3	3
***Tylototriton (Tylototriton) panwaensis***	3	3
*Tylototrition (Tylototriton) podichthys*	3	3
*Tylototrition (Tylototriton) shanjing*	13	9
***Tylototrition (Tylototriton) shanorum***	6*	4
*Tylototrition (Tylototriton) taliangensis*	10	10
***Tylototrition (Tylototriton) uyenoi***	13*	9
*Tylototrition (Tylototriton) verrucosus*	17*	14
*Tylototrition (Yaotriton) vietnamensis*	11	8
*Tylototrition (Yaotriton) wenxianensis*	7	5
*Tylototrition (Tylototriton) yangi*	2^+^	–
*Tylototrition (Yaotriton) ziegleri*	10	5
*Pleurodeles waltl*	5	5
*Chelotriton* Enspel	–	7
*Chelotriton* Randeck	–	1

* Species where pictures were additionally taken from literature.

^+^ Species where only life specimens were available. Species where holotype and/or paratype material is also included are given in bold.

### 2.1 Landmark data acquisition

**FIGURE 2:**
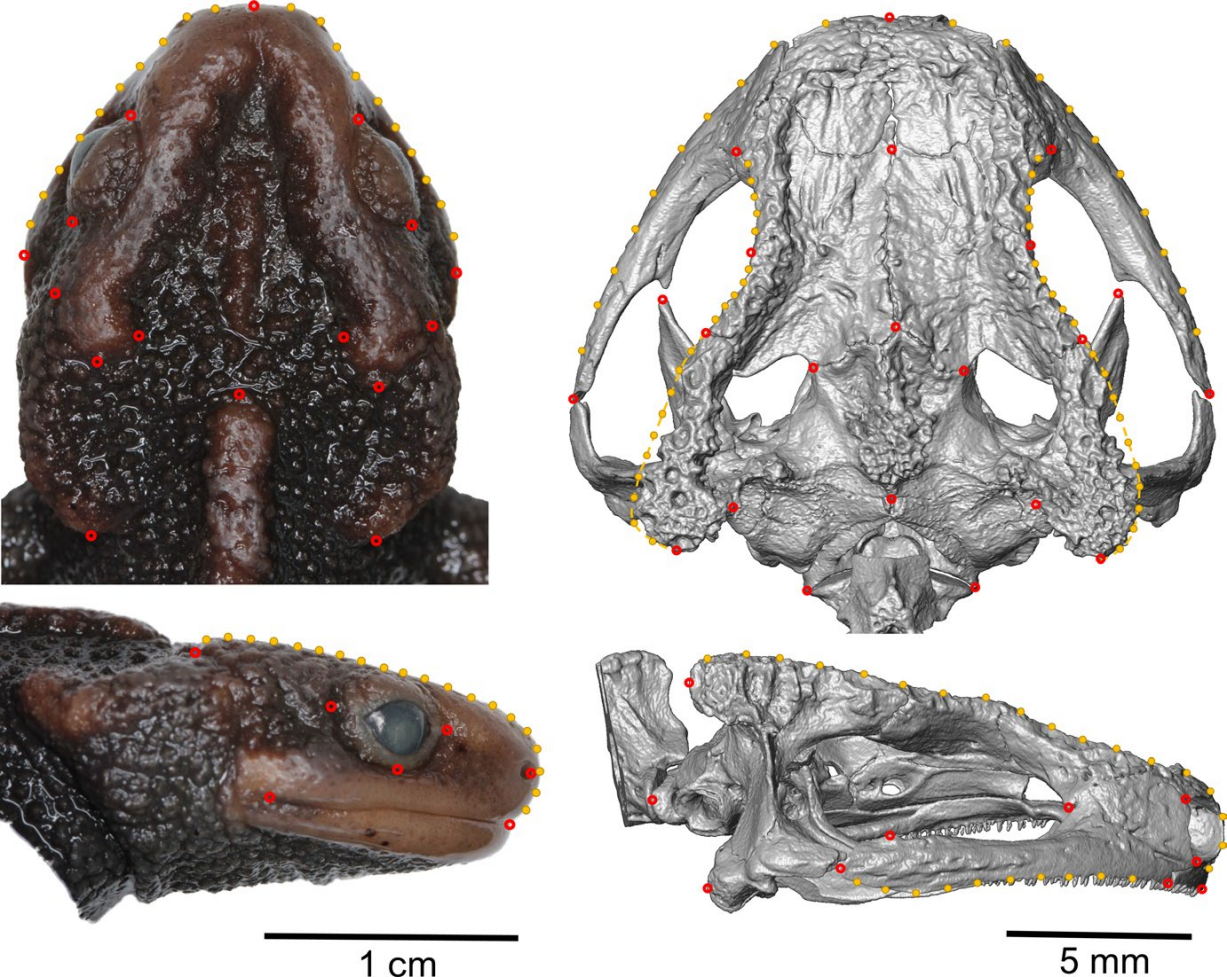
Landmark configurations in dorsal (top) and lateral (bottom) view of the external (left) and osteological (right) cranial morphology used in 2D geometric morphometric analyses of cranial shape of pleurodeline newts. Red circles denote landmarks, yellow dots semilandmarks

We investigated the cranial shape of pleurodeline salamanders by 2D geometric morphometric (GM) approaches. Two‐dimensional analysis was preferred over three‐dimensional analysis as the crania of fossils newts were too flat to apply 3D GM for comparison between extant and extinct samples (see below). First, we took standardized pictures of the extant representatives in dorsal and right lateral view of the cranium (Figure 2). As we could not access all representative species, we further searched in the literature for suitable images of crocodile newts. Figures from published work were extracted for *E. maxiquadratus* from Hou et al.*.* (2014), *Tylototriton anguliceps* from Le et al.*.* (2015), *Tylototriton broadoridgus* and *Tylototriton liuyangensis* from Yang et al.*.* (2014), *Tylototriton ngarsuensis* from Grismer et al.*.* (2018), *Tylototriton notialis* from Stuart et al.*.* (2010), a topotypic *Tylototriton verrucosus*, holotype of *Tylototriton shanorum* and *Tylototriton uyenoi* from Nishikawa et al.*.* (2014) and *Tylototriton  kachinorum* from Zaw et al.*.* (2019).

Second, extant and fossil specimens were scanned via µCT to allow investigation of the cranial skeleton. Scans were carried out either with a Bruker SkyScan1272 or within the X‐ray imaging laboratory at the Institute for Photon Science and Synchrotron Radiation, Karlsruhe Institute of Technology (KIT), employing a microfocus X‐ray tube (XWT‐225, X‐RAY WorX) and a flat panel detector (XRD 1621 CN14 ES, Perkin Elmer) in combination with a custom‐designed mechanical sample manipulator. For the datasets measured at KIT, octopus 8.6 (Inside Matters) was used to perform the tomographic reconstruction. Due to the time‐consuming procedure of µCT scanning, only a subsample per species was scanned (Table 1, Supporting Information Table S1). In some species with larger distribution areas or presumably different morphologies among localities, additional specimens were analyzed via µCT. In total, 121 specimens including fossils were µCT‐scanned (Table S1). The scan resolution for extant newts was either 20.1 (SkyScan) or 21.3 µm (KIT‐scanner). *Chelotriton* specimens were scanned at 35.2 µm resolution. Three‐dimensional reconstructions were processed in amira® 6.5 (Visualisation Science Group). Flattened and distorted during fossilization, *Chelotriton* specimens did not allow sufficient reconstruction in a three‐dimensional space. Nevertheless, to reconstruct a morphology which is most likely to represent its original dorsal shape, retrodeformation by algorithmic symmetrization using the software IDAV landmark editor v.3.7 (http://graphics.idav.ucdavis.edu/research/EvoMorph) was performed to reduce asymmetrical distortion in the fossil crania of the Enspel specimens (Tallman et al., 2014). Landmark configurations for retrodeformation were specifically adapted to each single fossil specimen, as deformation was different in each specimen. We also employed several retrodeformations with different sets of landmarks to receive results appearing as symmetrical as possible but simultaneously not diverging too much from the original shape (Supporting Information Figure S1). The only sample from Randeck Maar appears to be symmetrical and was therefore not retrodeformed. Two‐dimensional images of skulls were taken in dorsal view and, additionally, skull images of extant taxa were taken in the right lateral view (Figure 2) to allow comparison with external morphology. In the following, ‘head shape/morphology’ will refer to external cranial morphology including soft tissue, ‘skull shape/morphology’ to the osteology, and ‘cranial shape/morphology’ more generally to cranial morphology irrespective of the dataset analyzed herein.

For the analyses of head morphology, we digitized 16 landmarks and 20 semilandmarks in dorsal view and seven landmarks and 20 semilandmarks in lateral view. On skull images, 22 landmarks and 60 semilandmarks in dorsal view and 10 landmarks and 20 semilandmarks in lateral view were digitized (Figure 2, Supporting Information Table S2). Landmark digitization was carried out by one author using tpsUtil and tpsDig (Rohlf, 2016a; 2016b). Specimens were randomly shuffled. To test for accuracy of landmark placement, each landmark configuration was tested by digitizing one specimen five times and five other specimens of the same species. Procrustes distance to the mean shape of replicates and interindividuals were tested against each other to test whether intraindividual landmark placement was consistent in comparison with landmark placement between different individuals.

### 2.2 Geometric morphometrics

Two‐dimensional geometric morphometrics analysis was performed in R version 3.5.3 (R Development Core Team, 2019) using the packages geomorph v.3.1.1 and RRPP v. 0.4.1 (Collyer and Adams, 2018; Adams et al., 2019). The procedure of analysis was equal for each dataset. Missing landmarks were estimated by applying thin plate spline approach using the function ‘estimate.missing’, as complete landmark configurations are needed for subsequent procedures. The estimation of missing landmarks was done separately for extant and extinct members in order not to mix up shape variation. In extant specimens in total, three landmarks in two specimens were missing only, whereas in fossils, 105 of 672 landmarks were missing due to locally unsuitable preservation. Three fossils had preserved the entire landmark configuration. Two fossil specimens accumulated most of the missing landmarks, one having 50 and 44, respectively, missing landmarks comprised mainly of semilandmarks. A generalized Procrustes alignment (GPA) was employed with the function ‘gpagen’ to remove variation due to location, rotation and scale of the samples (Rohlf and Slice, 1990). Simultaneously, semilandmarks were slid by minimizing bending energy (Bookstein, 1997a; Perez et al., 2006). This resulted in a new dataset of so‐called Procrustes coordinates of each landmark and centroid size (CS) for each sample. Centroid size is a measure of scale in geometric morphometrics being independent of shape and is calculated as the square root of the summed squared distances of each landmark from the centroid (Bookstein, 1997b; Zelditch et al., 2012). Ivanović and Arntzen (2017) showed that the allometric shape component explains a relatively low amount of shape variation within Salamandridae and even less within pleurodeline newts. Thus, we removed allometry, which is beyond the scope in this study, from the datasets to emphasize other potential sources of variation. Allometry‐free shapes were generated by transforming the residuals from multivariate regression of shape to log(CS) using the generic function ‘procD.lm’ and applying these to the mean shape values. Allometry‐free shapes were used to explore cranial shape. First, a principal component analysis (PCA) was performed on the covariance matrix of the Procrustes shape coordinates with the function ‘plotTangentSpace’. To test the effect of species and genus on cranial shape and log(CS), we performed a Procrustes analysis of variance (ANOVA) using the function ‘procD.lm’. A pairwise comparison of species and genera was carried out *post hoc* to clarify which species and genera were different from each other. Species with only one sample were excluded from *post hoc* testing. Alpha level for multiple testing was adjusted via Bonferroni correction.

**FIGURE 3:**
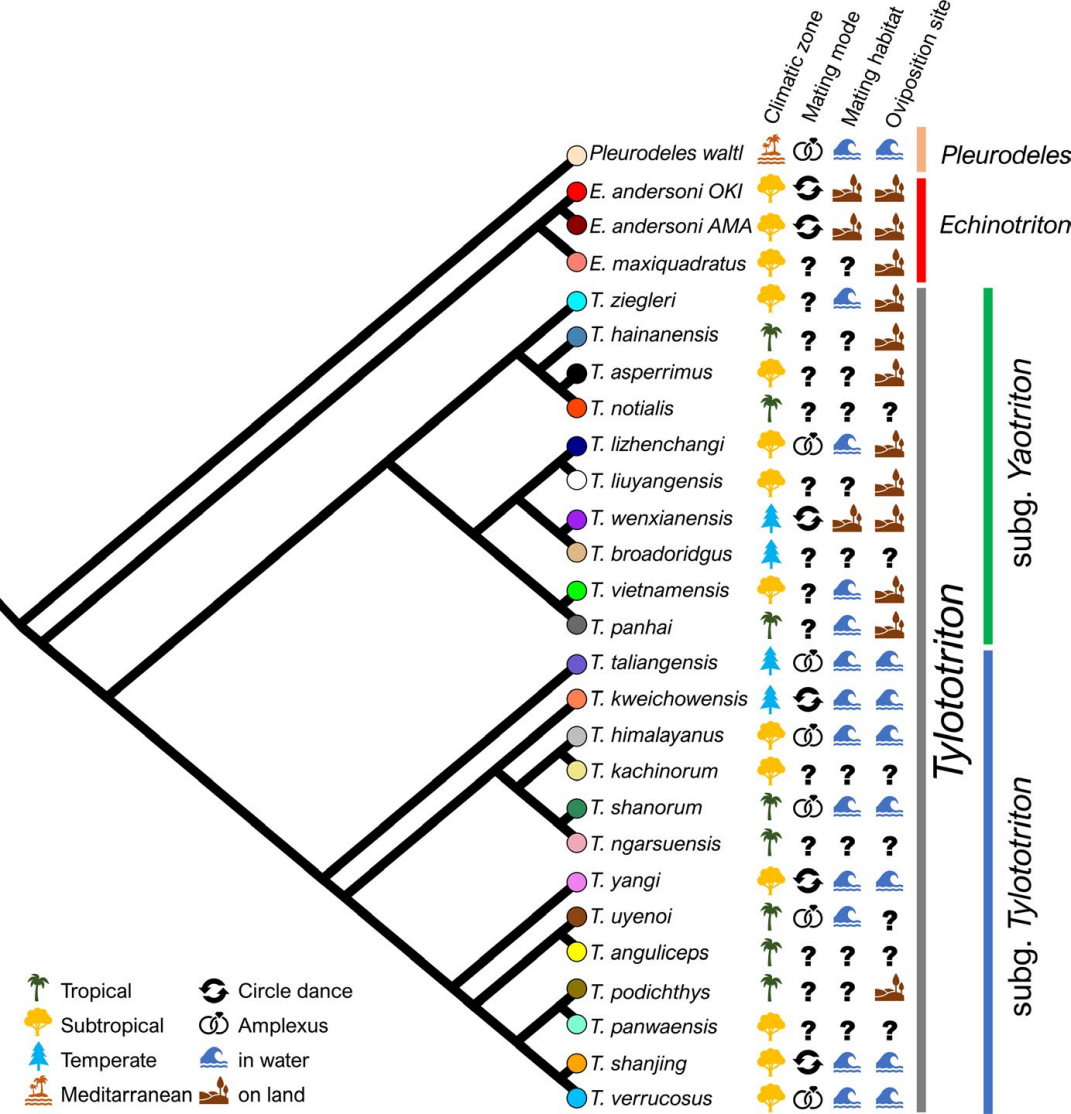
Phylogeny of pleurodeline newts including the genera *Pleurodeles, Echinotriton* and *Tylototriton* following Zaw et al., 2019. Data on distribution in climatic zones and life history traits (mating mode, mating habitat, oviposition site) are illustrated. Color codes of tree tips correspond to settings in subsequent figures

For further analysis, species means were calculated from Procrustes coordinates and for log(CS). Again, a PCA was conducted on the species’ mean shapes. Visualization including phylogeny was performed using the function ‘plotGMPhyloMorphoSpace’, creating a plot of principal components for a set of Procrustes coordinates. Internal nodes were calculated by the squared‐changed parsimony method (Rohlf, 2002; Klingenberg and Gidaszewski, 2010). We tested whether cranial shape was affected by phylogeny using the function ‘physignal’ on different taxonomic levels within pleurodeline newts. This function estimates the phylogenetic signal using the K*mult* statistics, assuming the Brownian motion model of evolution (Adams, 2014a). We followed the most recent phylogeny of Zaw et al. (2019), including all species used in this study (Figure 3). Contrary to Fei et al. (2012), we treated *Tylototriton kweichowensis* and *T. taliangensis* as members of the subgenus *Tylototriton* and did not follow the concept of recognizing additional subgenera accommodating the species. Evolutionary divergence for the two populations of *E. andersoni* from Okinawa and the Amami archipelago was taken from Kurabayashi et al. (2012). Skull shape in dorsal view was analyzed once without and once with the *Chelotriton* datasets for two reasons: to allow a better comparability of datasets for external morphology and osteology in extant newts and to help examine whether fossil specimens potentially add variation to the morphospace in a particular direction, and hence affect the morphospace of the extant members (Pérez‐Ben et al., 2019).

Finally, we tested whether cranial morphology correlates with ecological and reproductive biology via phylogenetic ANOVA (Procrustes ANOVA and regression models in a phylogenetic context assuming the Brownian motion model of evolution) using the function ‘procD.pgls’ (Adams, 2014b). We collected available data in the literature on the following traits: mating mode (amplexus, mating dance), mating habitat (terrestrial, aquatic) and oviposition site (terrestrial, aquatic) (Figure 3). Further, species distribution area was assigned to one of the following main biomes: tropical, subtropical, temperate, Mediterranean according to Kottek et al. (2006) and Woodward et al. (2004) (Figure 3). Significance testing was performed by permutation procedures with 10,000 iterations implemented in the RRPP package (Collyer and Adams, 2018; Adams et al., 2019).

## 3 RESULTS

### 3.1 Lateral head morphology

**FIGURE 4:**
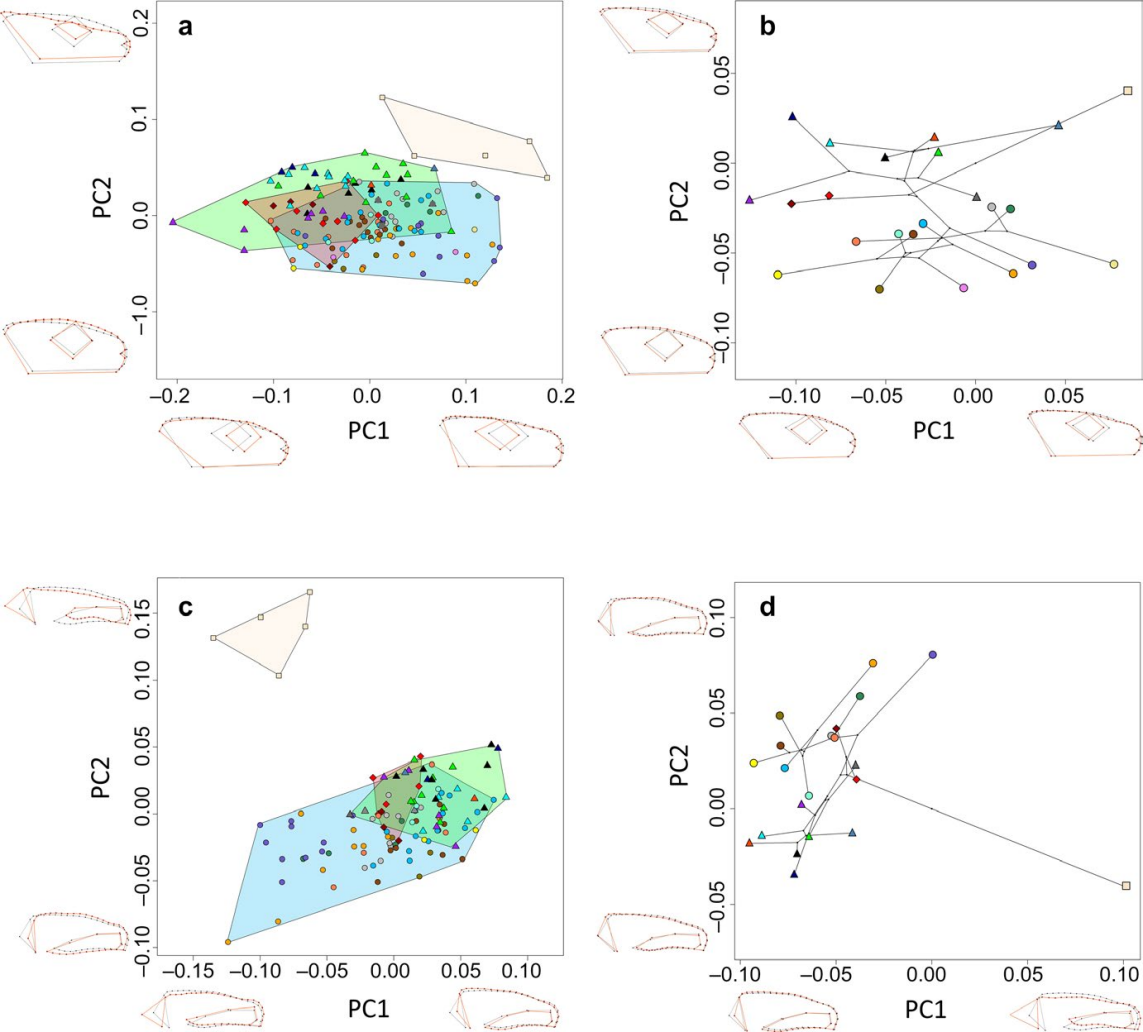
PCA plots of GPA‐aligned, allometry‐free shapes of cranial morphology in lateral view of pleurodeline newts. Black wireframe corresponds to the mean shape, red wireframe represents the shape at the extreme value of the respective PC axes. (a) External morphology of all specimens. (b) External morphology of species mean shapes. (c) Osteology of all specimens. (d) Osteology of species mean shapes. Triangles correspond to the members of the subgenus *Yaotriton*, ellipses to subgenus *Tylototriton*, diamonds to *Echinotriton*, and square to *Pleurodeles.* For color code, see Figure 2

In terms of head morphology, principal component (PC) 1 explained 55.3% and PC2 14.2% of the observed variance. Ribbed newts were well separated from the crocodile newts, whereas the latter largely overlapped in their morphospace (Figure 4a). *Echinotriton andersoni* from Okinawa and Amami were not separated. Positive PC1 scores were associated with a posterior eye position and labial angle. Further, the naris was positioned more dorsally, and the dorsolateral ridge ended at a more anterior and dorsal position, leading to a straight occiput line connecting the most posterior end of the dorsolateral ridge and labial angle. Negative PC1 scores comprised an anterior position of the eye and labial angle with a more ventrally positioned naris and posterior end of the dorsolateral ridge. The occiput was more diagonal. High loadings of PC2 were associated with a flattened head with a straight cranial roof and an anterior shift of the occiput, whereas negative PC 2 values were associated with a dorsoventrally raised head with a convex cranial roof above the orbit and a posteriorly shifted occiput.

In the analysis on species means, PC1 explained 60.3% and PC2 18.6% of the variance. The morphological changes along PC axes were similar, as described above, but less pronounced (Figure 4b). The subgenera *Yaotriton* and *Tylototriton* were morphologically well separated in the morphospace along the second PC axis. *Echinotriton* and *Pleurodeles* morphologically resembled *Yaotriton* more than *Tylototriton*.

In skull morphology, PC1 explained 31.0% and PC2 22.6% of the observed variance. *Pleurodeles* occupied a separate area in the morphospace along the PC2 axis, whereas *Echinotriton* and *Tylototriton* overlapped (Figure 4c). Positive PC1 scores were associated with a slender snout tip, a ventrally moved maxillary tip, a lower dorsolateral ridge and a shorter but higher occiput, which is tilted forward. Negative PC1 scores were associated with a bulkier snout tip, dorsally moved maxillary tip and dorsolateral ridge, and a posterior elongated occiput which is orientated almost perpendicularly. Positive values on the PC2 axis were associated with an uplift and shortening of the maxillary, an elongated snout tip, a flatter skull roof slightly elongated posteriorly and a backward tilted occiput with posteriorly moved occipital condyles.

Morphospace on species means was rotated along both axes, PC1 (42.1%) and PC2 (28.7%). *Pleurodeles* occupied the extreme value along PC1 and both subgenera *Yaotriton* and *Tylototriton* again were distinct. Whereas *E. andersoni* from Okinawa was closer to *Yaotriton,* specimens from the Amami archipelago fell within the morphospace of the subgenus *Tylototriton* (Figure 4d). Positive PC1 scores were associated with a slightly bulkier snout tip, a flatter skull, a shorter and uplifted maxilla, a perpendicular occiput and posteriorly shifted occipital condyle. Negative PC1 scores were associated with an only slight elongation of the maxillary and an anteriorly moved occipital condyle. Positive scores on PC2 were associated with an elongation of the maxilla, a perpendicular orientation of the occiput, an elevation of the anterior skull roof and a drop of the posterior end of the dorsolateral ridge. Negative scores on PC2 were associated with a shortening of the maxilla, a slight backward tilt of the occiput, an uplift of the posterior end of the dorsolateral ridge and a slight drop of the anterior skull roof.

### 3.2 Dorsal head morphology

**FIGURE 5:**
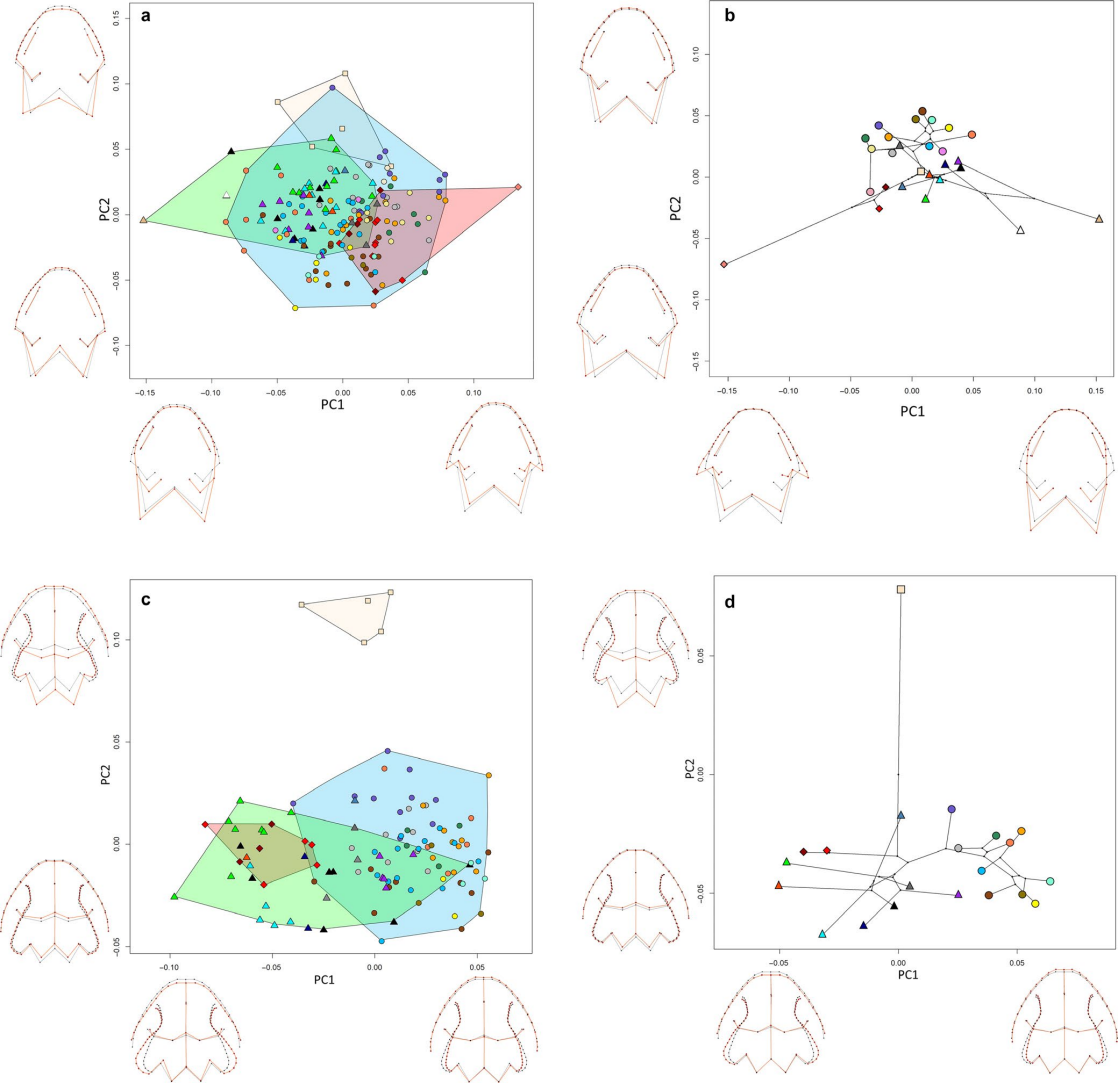
PCA plots of GPA‐aligned, allometry‐free shapes of cranial morphology in dorsal view of pleurodeline newts excluding *Chelotriton*. Black wireframe corresponds to the mean shape, red wireframe represents the shape at the extreme value of the respective PC axes. (a) External morphology of all specimens. (b) External morphology of species mean shapes. (c) Osteology of all specimens. (d) Osteology of species mean shapes. Triangles correspond to the members of the subgenus *Yaotriton*, ellipses to subgenus *Tylototriton*, diamonds to *Echinotriton* and square to *Pleurodeles.* For color code see Figure 2

In dorsal view, PCA on head morphology explained 25.6% on PC1 and 15.3% on PC2 of the observed variance. Although *Pleurodeles* occupied the morphospace at the end of PC2, it still largely overlapped with the morphospace of *Tylototriton* subgenera (Figure 5a). *Pleurodeles* was best separated from crocodile newts along a gradient of PC2 and PC4 (10.1%, Supporting Information Figure S2). *Tylototriton* subgenera overlapped with both *Yaotriton* and *Echinotriton*, covering almost the entire morphospace of pleurodeline newts (Figure 5a). *Echinotriton andersoni* overlapped with both *Tylototriton* subgenera, whereas *E. maxiquadratus* was well separated. This was also the case for *Tylototriton* (*Yaotriton*) *broadoridgus* at the other side of the morphospace. There was no clear separation of Okinawa and Amami populations. With positive scores of PC1, head morphology was associated with anterior parotoid tips, slender and anteriorly moved dorsolateral ridges, slightly prolonged snout, a wider cranium and a more posterior widest head width. Negative PC1 scores were associated with posterior shift of parotoid tips and dorsolateral ridges, the latter one also being wider, having a shorter snout and a slender cranium with a more anterior position of the largest head width. Positives scores on PC2 were associated with a truncated snout, posterior position of eyes and of the vertebral ridge, and distally shifted parotoid tips. Negative scores were associated with a pointier snout tip, anteriorly shifted eyes and end of vertebral ridge and proximal parotoid tips.

In the morphospace of species means, PC1 explained 48.8% and PC2 16.9% of the variance. Subgenera *Tylototriton* and *Yaotriton* were well separated except for *T. (Y.) panhai* falling into the morphospace of subgenus *Tylototriton* (Figure 5b). *Echinotriton* morphospace was well separated from *Tylototriton,* with only a slight overlap. *Echinotriton maxiquadratus* fell far apart from *Tylototriton* compared with *E. andersoni*. *Tylototriton (Y.) liuyangensis* and *T. (Y.) broadoridgus* also fell far apart from their other congeners. *Pleurodeles* fell right into the middle of the occupied morphospace. Ribbed newts were separated well from crocodile newts along PC3, explaining 12.2% of variance (Supporting Information Figure S3). Positive scores on PC1 were associated with posterior shift of parotoid tips and dorsolateral ridges and wider dorsolateral ridges. Negative PC1 scores were associated with anterior shifts of the parotoid tips and dorsolateral ridges, narrower ridges and a wider head. Positive scores of PC2 were associated with a slight elongation and narrowing of the cranium and a proximal shift of parotoid tips, whereas negative PC2 scores were linked to a truncation of the snout, widening of the cranium and a distal shift of the parotoid tips. Additionally, positive PC3 scores were associated with a posterior and proximal movement of eyes, an anterior shift of the widest head position and a posterior shift of the terminal point of the vertebral ridge. Negative PC3 scores were related to an anterior shift of the terminal point of the vertebral ridge. Otherwise, morphological changes were only marginal.

PC1 explained 23.6% and PC2 explained 22.1% of the observed variance in osteology. Again, *Pleurodeles* were well separated from all other groups (Figure 5c). The subgenera of *Tylototriton* overlapped within about half of their respective morphospace. *Echinotriton* settled within the space of *Yaotriton*, only slightly overlapping with subgenus *Tylototriton*. Positive scores on PC1 were associated with an elongation of the snout tip and maxilla, the anterior shift of orbit edges and slender dorsolateral ridges. Negative PC1 scores were related to the shortening of the snout tip and maxilla, a posterior shift of the orbit edges and widening of dorsolateral ridges. Positive scores of PC2 were associated with a protruding and blunter snout, a shorter maxilla, a posterior shift of the fronto‐parietal suture, a posterior shift of the occipital condyles and parietals, slender dorsolateral ridges and a slender cranial roof between orbits. Negative PC2 scores were associated with a slightly truncated snout and an anterior shift of the fronto‐parietal suture, occipital condyles and parietals.

PC1 of species mean shapes explained 27.9% and PC2 25.9% of morphospace variance. Notably, *Pleurodeles* appeared far apart from all crocodile newts but also *Yaotriton* and *Tylototriton* were well separated without overlapping (Figure 5d) and *Echinotriton* resembled *Yaotriton* more than species of subgenus *Tylototriton.* The morphological changes along PC axes were similar to those described before.

### 3.3 Morphology of fossil *Chelotriton*

**FIGURE 6:**
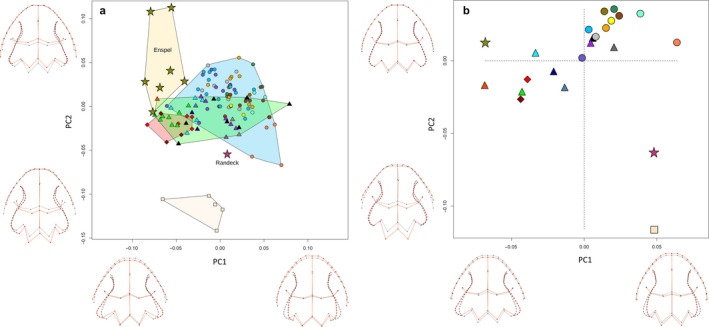
PCA plots of GPA‐aligned, allometry‐free shapes of osteological, cranial morphology in dorsal view of pleurodeline newts including *Chelotriton*. Black wireframe corresponds to the mean shape, red wireframe represents the shape at the extreme value of the respective PC axes. (a) PCA plot for all specimens. (b) PCA on species mean shapes. Triangles correspond to the members of the subgenus *Yaotriton*, ellipses to subgenus *Tylototriton*, diamonds to *Echinotriton*, the square to *Pleurodeles*, stars to *Chelotriton.* For color code, see Figure 2

When including fossil crocodile newts, PC1 explained 23.6% and PC2 22.1% of the variance. *Chelotriton* of the two deposits occupied different positions in the morphospace. *Chelotriton* from Enspel were located at the upper left quadrant, whereas *Chelotriton* from Randeck fell into the lower right quadrant (Figure 6a). Neither lay within the morphospace of extant taxa, whereas *Pleurodeles* was morphologically distinct and occupied a different part of the morphospace. Morphological changes along PC1 axis were similar to the skull dataset excluding *Chelotriton* described above. The second PC axis changed orientation, positive scores comprised an anterior shift of the fronto‐parietal suture and the occiput, prolonged maxilla, a wider skull roof between orbits and more laterally orientated pterygoids. Negative scores along PC2 comprised a shortening of the maxilla, posterior shift of the fronto‐parietal suture, an elongation of the occiput including the dorsolateral ridges and a narrower skull roof between orbits.

On species means, PC1 explained 27.9% and PC2 25.9% of the observed variance. The subgenera *Yaotriton* and *Tylototriton* were also well separated, although *Tylototriton (Tylototriton) taliangensis* fell within the morphospace of *Yaotriton* (Figure 6b). Again, neither of the fossil *Chelotriton* fell into the morphospace of *Tylototriton*, *Echinotriton* or *Pleurodeles*. Specimens from Enspel were located closer to *Yaotriton*, whereas the Randeck specimen was placed inbetween *Tylototriton* subgenera and *Pleurodeles*. Morphological changes along PC axes were similar to those described before, with the exception that positive PC1 scores additionally comprised a slight anterior shift of the fronto‐parietal suture and occiput, and the opposite was true for negative scores.

### 3.4 Phylogeny, ecology and shape

In all datasets, a phylogenetic signal was present (Table 2. The influence of phylogeny was still strong within the genus *Tylototriton*, whereas within its subgenera it was only detectable among subgenus *Tylototriton,* and only in dorsal morphology. Shape differed interspecifically and generically in all datasets (see Table 3). Post‐hoc testing revealed that all pleurodeline newt genera were morphologically distinct in cranial shape (Supporting Information Tables S2, S6, S14 and S18). Only in lateral skull view was no difference among *Tylototriton* and *Echinotriton* detected, whereas *Pleurodeles* showed a distinct cranial morphology from all other pleurodeline newts (Supporting Information Table S10). The two populations of *E. andersoni* were different neither in cranial shape or size. In lateral view, both *Echinotriton* populations were less distinct in their morphology from *Tylototriton* (Supporting Information Tables S1 and S9). In dorsal view, especially *E. andersoni* from Okinawa appeared morphologically distinct from many *Tylototriton* species (Supporting Information Tables S5 and S13). *Yaotriton* subgenera showed only little interspecific differences in various datasets, whereas divergence within *Tylototriton* subgenera was more marked (Tables S1, S5, S9 and S13). In particular, *T. (T.) kweichowensis* and *T. (T.) taliangensis* were distinct from several consubgeners. Within *Yaotriton*, *T. (Y.) vietnamensis* and *T. (Y.) ziegleri* were highly divergent from *Tylototriton* subgenera, but *T. (T.) shanjing* and *T. (T.) taliangensis* appeared morphologically distinct from most *Yaotriton* species.

**TABLE 2:** Test for phylogenetic signal in 2D morphometrics cranial datasets of Pleurodelini, genus *Tylototriton* and the two subgenera *Yaotriton* and *Tylototriton*. Significant *p*‐values are given in bold

Pleurodelini	*K*	*p*
Head lateral	.363	**.0039**
Head dorsal	.428	**.0048**
Skull lateral	.51	**.0035**
Skull dorsal	.52	**<.0001**
Genus *Tylototriton*		
Head lateral	.55	**.035**
Head dorsal	.58	**.0073**
Skull lateral	.46	.2
Skull dorsal	.69	**<.0001**
Subgenus *Tylototriton*		
Head lateral	.59	.026
Head dorsal	.73	**.025**
Skull lateral	.48	.38
Skull dorsal	.69	**.013**
Subgenus *Yaotriton*		
Head lateral	.68	.5
Head dorsal	.64	.63
Skull lateral	.68	.37
Skull dorsal	.7	.36

**TABLE 3:** Procrustes ANOVA of 2D morphometric shape data and centroid size (CS) of crania of pleurodeline newts tested for species and genus. Significant *p*‐values are given in bold

	Model	*df*	*F*	*p*
Head lateral	shape ~ species	19	7.6	**<.0001**
	shape ~ genus	2	11.7	**<.0001**
	log(CS) ~ species	19	14.0	**<.0001**
	log(CS) ~ genus	2	.3	.78
Head dorsal	shape ~ species	22	5.7	**<.0001**
	shape ~ genus	2	12.9	**<.0001**
	log(CS) ~ species	22	1.7	**<.0001**
	log(CS) ~ genus	2	6.4	**.0021**
Skull lateral	shape ~ species	18	6.4	**<.0001**
	shape ~ genus	2	14.2	**<.0001**
	log(CS) ~ species	18	11.8	**<.0001**
	log(CS) ~ genus	2	.04	.96
Skull dorsal	shape ~ species	18	7.7	**<.0001**
	shape ~ genus	2	13.5	**<.0001**
	log(CS) ~ species	18	11.4	**<.0001**
	log(CS) ~ genus	2	.8	.45
Skull dorsal incl. *Chelotriton*	shape ~ species	19	8.6	**<.0001**
	shape ~ genus	3	15.5	**<.0001**
	log(CS) ~ species	19	24.0	**<.0001**
	log(CS) ~ genus	3	34.6	**<.0001**

Fossil *Chelotriton* from Enspel (the single *Chelotriton* from Randeck was excluded due to sample size) exhibited a cranial morphology distinct from nearly all other pleurodeline newts (Supporting Information Table S17). Centroid size differed interspecifically in all datasets but on the genus level, only head and skull morphology including *Chelotriton* was different (Table 3). Post hoc tests revealed that the dorsal head morphology of *Tylototriton* was smaller than in *Echinotriton* (Supporting Information Table S4). Further, *Chelotriton* was larger than all other genera and species of pleurodeline newts except *T. (T.) kweichowensis* (Supporting Information Tables S19 and S20). In dorsal view, populations from Okinawa of *E. andersoni* tended to be larger than many *Tylototriton*. Only *T. (T.) kweichowensis* developed a similar cranial size, being even larger than *E. andersoni* from the Amami archipelago and most other congeners (Supporting Information Tables S3, S7, S11 and S15). Within *Yaotriton, T. (Y.) asperrimus* had the largest cranial size, but only *T. (Y.) vietnamensis* and *T. (Y.) wenxianensis* were consistently smaller among the datasets. Within *Tylototriton* subgenera, mainly *T. (T.) kweichowensis* diverged in cranial size from its consubgeners. Laterally, *T. (T.) taliangensis* diverged from *T. (T.) uyenoi* and *T. (T.) yangi* in cranial size, whereas the centroid size of *Pleurodeles* was smaller than in *T. (T.) kweichowensis* in lateral view, but larger than in *T. (Y.) lizhenchangi* and *T. (Y.) wenxianensis* in dorsal head morphology.

**FIGURE 7:**
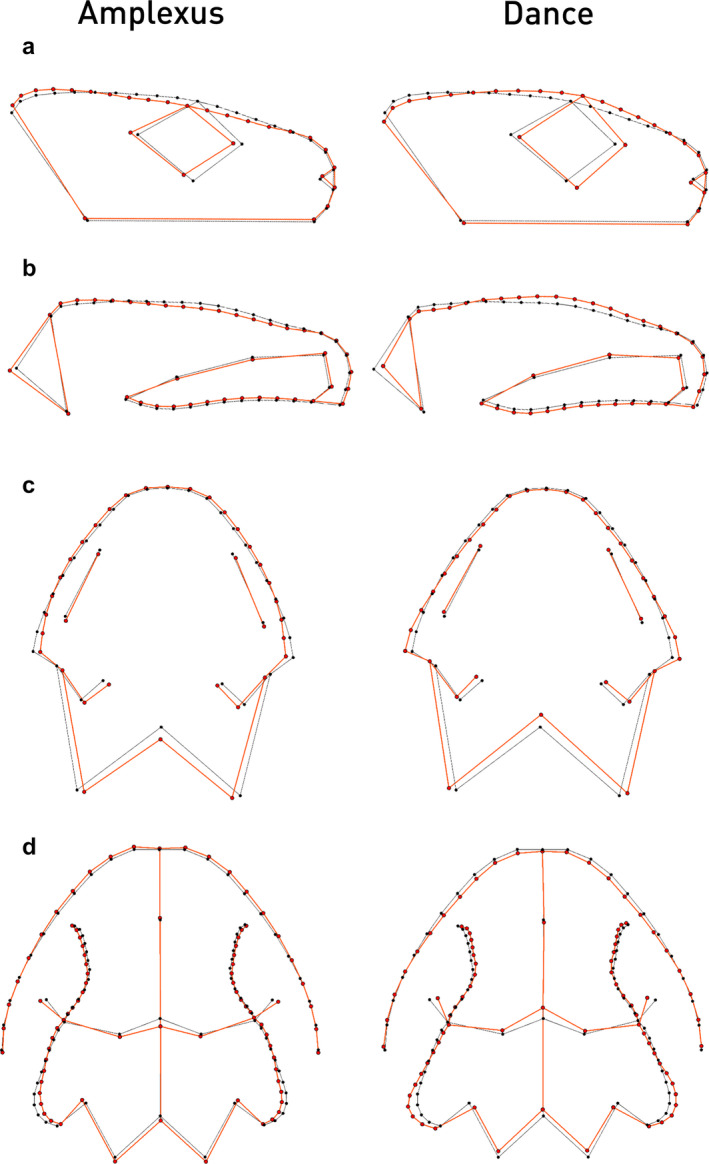
Shape change of cranial morphology in different datasets of 2D morphometric shape data of pleurodeline newts corresponding to different mating modes. (a) Lateral head morphology, (b) lateral skull morphology, (c) dorsal head morphology, (d) dorsal skull morphology. Black wireframe corresponds to the mean shape, red wireframe represents the shape of either amplectant species (left column) or dancing species (right column). Shape changes are magnified by a factor of two

Only mating mode was associated with the cranial shape in three of four datasets accounting for phylogeny (Table 4). In lateral morphology, species using an amplexus exhibited a smaller eye diameter, the eye being also slightly posteriorly shifted. The cranial roof was flatter in those species, whereas the posterior end of the dorsolateral bony ridges was elevated (Figure 7a,b). Species using a mating dance for copulation in general exhibited a bulkier cranium. The eye diameter was enlarged and anteriorly shifted. The cranial roof was elevated and the dorsolateral bony ridges were inclined. Furthermore, the occiput was shorter in those species. In dorsal view, main shape differences comprised more slender dorsolateral bony ridges and posteriorly moved pterygoid tips, occiput and midcranial suture among frontals and parietals in amplectant species (Figure 7c,d). In dancing species, the opposite shape changes were observed.

**TABLE 4:** Procrustes ANOVA in a phylogenetic framework of 2D morphometric shape datasets of crania of pleurodeline newts tested for ecological traits. Significant *p*‐values are given in bold

	Model	*df*	*F*	*p*
Head lateral	shape ~ mating mode	1	3.61	**.044**
	shape ~ mating habitat	1	1.61	.199
	shape ~ oviposition site	1	1.74	.17
	shape ~ climate	3	.65	.67
Head dorsal	shape ~ mating mode	1	.96	.39
	shape ~ mating habitat	1	.46	.76
	shape ~ oviposition site	1	1.22	.27
	shape ~ climate	3	1.48	**.012**
Skull lateral	shape ~ mating mode	1	8.41	**.011**
	shape ~ mating habitat	1	.34	.075
	shape ~ oviposition site	1	1.12	.3
	shape ~ climate	3	.92	.44
Skull dorsal	shape ~ mating mode	1	3.58	**.014**
	shape ~ mating habitat	1	.61	.72
	shape ~ oviposition site	1	1.3	.25
	shape ~ climate	3	1.03	.38
Skull dorsal incl. *Chelotriton* ^a^	shape ~ mating mode	1	2.67	**.041**
	shape ~ mating habitat	1	.41	.88
	shape ~ oviposition site	1	1.29	.26
	shape ~ climate	3	.96	.5

^a^ Note that *Chelotriton*‐shape *per se* is not included in the models of LH traits, as no information on these is available for this genus. The models instead concern data processing (GPA alignment) of the remaining shapes prior analysis.

## 4 DISCUSSION

### 4.1 Morphology and phylogeny

We investigated the cranial morphology of ribbed and crocodile newts in an integrative approach including their external head and skull morphology. To shed more light on the evolutionary history of pleurodeline newts, well‐preserved fossil specimens of the closely related genus *Chelotriton* were included in our multivariate analyses of cranial morphology in relation to selected reproductive traits and distribution across biomes.

General shape changes in external and osteological morphology were similar. Correlations of soft and hard tissue morphometrics were shown already in another basal salamandrid salamander (Pogoda and Kupfer, 2018). However, osteology provides many more possibilities for placing precise landmarks, likely representing a better basis for evolutionary research. Ribbed newts were well separated from crocodile newts in various morphospaces previously confirmed by cranial three‐dimensional morphometric analysis (Ivanović and Arntzen, 2017). Cranial shape differentiation coincides with the spatial distribution patterns, Mediterranean *Pleurodeles* being differentiated from the Asian *Echinotriton* and *Tylototriton*. Nevertheless, mean shapes of the latter genera were different in all except but one of the datasets. To increase support for these results, it is suggested to add more specimens of *E. chinhaiensis* and *E. maxiquadratus*. Dubois and Raffaelli (2009) described the subgenus *Yaotriton* including the uniformly ‘black’‐coloured crocodile newts of the *asperrimus* group. Later, another two subgenera were distinguished by Fei et al. (2012): i.e. for *T. kweichowensis* the monophyletic subgenus *Qiantriton* and for *T. taliangensis* and *T. pseudoverrucosus Liangshantriton* were suggested. The latter is recognized by some authors (Gong et al., 2018) but this would classify *Tylototriton* as nonmonophyletic and hence is not supported by the majority of the community (Frost, 2018). We showed that *T. (T.) kweichowensis* and *T. (T.) taliangensis* show some degree of morphological distinctiveness from the other members of the subgenus *Tylototriton*. This may explain the phylogenetic signal within *Tylototriton* subgenera in terms of dorsal morphology, as those two species are phylogenetically at the base of the ancestral line. Overall, *T. kweichowensis* and *T. taliangensis* do not occupy a separate morphospace and thus are still treated as members of *Tylototriton* subgenera. A clear separation of *Yaotriton* and *Tylototriton* subgenera (*verrucosus* group) is only achieved when species mean shapes are considered. In lateral view, species of *Yaotriton* have a flatter cranium and a steeper occiput with a shorter maxilla, compared with *Tylototriton* subgenera. In dorsal view, *Yaotriton* exhibits wider dorsolateral ridges, a shorter snout, maxilla and smaller orbits than members of the subgenus *Tylototriton*. *Echinotriton* resembles more the species of *Yaotriton* than *Tylototriton* subgenera, including a generally shared appearance of *Yaotriton* and *Echinotriton*: the latter exhibit only few orange highlighted body structures, e.g. tail edges, digits and parotoid tips, whereas species of *Tylototriton* subgenera are often more colorful (Nussbaum and Brodie, 1982; Hernandez, 2016). The particular color patterns likely correlate with a peculiar defence behavior, the ‘unkenreflex’, which is known only from *Echinotriton* and *Yaotriton* (Brodie et al., 1984; Sparreboom et al., 2001; Gong and Mu, 2008). Other antipredator postures were described for *T. (T.) verrucosus* (Brodie et al., 1984). However, to draw final conclusions about phylogenetic relationships on a larger scale, more data would be required.

The phylogenetic signal is strongest among *Tylototriton*, whereas within its subgenera we detected only an influence of phylogeny within subgenus *Tylototriton.* In the phylomorphospace, the closely related *T. (T.) anguliceps, T. (T.) uyenoi, T. (T.) podichthys* and *T. (T.) panwaensis* and also *T. (T.) himalayanus, T. (T.) shanorum, T. (T.) kachinorum* and *T. (T.) kweichowensis* plot together, whereas *T. (T.) podichthys/panwaensis* is sister to *T. (T.) verrucosus/shanjing* (Figures 4 and 5). The latter apparently diverged in the opposite direction of the phylomorphospace. The missing phylogenetic signal in lateral morphology is likely attributable to the low number of taxa included in the analysis. *Tylototriton (T.) verrucosus* and *T. (T.) shanjing* are two sister taxa, whereas the status of *T. (T.) shanjing* is still under debate (Zhang et al., 2007; Zhao et al., 2012). Although the genetic divergence is quite low among the two taxa (e.g. Phimmachak et al., 2015a; Wang et al., 2018; Grismer et al., 2019), most authors accept *T. (T.) shanjing* as a valid taxon (e.g. Stuart et al., 2010; Nishikawa et al., 2013) and the description of *T. (T.) pulcherrimus* adds to this confusion. Even though it was synonymized with *T. (T.) shanjing* by Nishikawa et al. (2013), other authors accept *T. (T.) pulcherrimus* as a valid taxon (Grismer et al., 2019; Zaw et al., 2019). Unfortunately, data for *T. (T.) pulcherrimus* were not available for this study. *Tylototriton (T.) shanjing* is distinguished from *T. (T.) verrucosus* mainly on differences in coloration (Nussbaum et al., 1995). In the phylomorphospace, *T. (T.) verrucosus* and *T. (T.) shanjing* are neither closer nor further apart from each other in comparison with other sister taxa within *Tylototriton*. Recently, Grismer et al. (2019) detected no morphometric differences between *T. (T.) verrucosus* and *T. (T.) shanjing*. In contrast, our pairwise comparisons indicated a more distinct morphology, especially in the lateral view of skulls (Table S9). As several populations, formerly assigned to *T. (T.) verrucosus* or *T. (T.) shanjing,* have been described as new species in recent years, more detailed fieldwork is needed, especially in northern Indochina, to identify new taxa and distribution boundaries.

*Chelotriton* from Enspel resembles more closely *Yaotriton* and *Echinotriton* than subgenus *Tylototriton* in the morphospace of PC1 and PC2. On other PC components up to PC9 (accounting for > 95% of variance), Enspel‐*Chelotriton* arrived mostly closer to *Echinotriton* (not shown)*.* As pairwise comparisons revealed a strong cranial disparity throughout extant pleurodeline newts, the phylogenetic position of *Chelotriton* from Randeck is far from clear. In morphospace, it falls between *Tylototriton* and *Pleurodeles* but on other PC components, Randeck‐*Chelotriton* arrived closer to *Tylototriton* subgenera than to *Pleurodeles* (not shown), indicative of the newt fossil remains of the two deposits belonging to different species (already assumed by Schoch et al., 2015). Obvious morphological differences are visible among *Chelotriton* specimens, e.g. a shorter maxilla and missing quadrate spines in the specimen from Randeck crater lake (Schoch et al., 2015). Analysis of different skull datasets, both including and excluding *Chelotriton,* showed weak differences in the morphospace of extant relatives and also revealed that *Chelotriton* does not add much additional variation to the morphospace. Thus, the analyses of fossil newts likely allow some general conclusions to be drawn. Nevertheless, one must always keep in mind that taphonomic processes could severely alter the shape of fossil crania. Most notably, the dorsolateral ridges are displaced distally, presumably altered by taphonomy. Further, morphological traits for landmark acquisition are frequently altered or destroyed, and estimating those by algorithms never can reproduce the full truth. *Chelotriton* specimens cluster well together in the morphospace and specimens with estimated missing landmarks are not clustered in a specific region of it. Hence, we assume that the estimation of the missing landmarks has not led to significant alteration of the data. More detailed morphological comparisons among *Chelotriton* specimens remain to be made, and the four currently known species within the genus await validation (Marjanović and Witzmann, 2015). Nevertheless, we have shown that *Chelotriton* represents the largest bodied members within pleurodeline newts, although extant members deviate only little in their cranial size. *Echinotriton andersoni* from the Amami archipelago is thought to be smaller in body size than the populations from Okinawa (Utsunomiya et al., 1978; Hernandez, 2016). Our study does not support the same pattern in cranial size, although this might be due to the small sample size of Amami specimens.

### 4.2 Morphology and ecology

Data on the general ecology are still scarce for crocodile newts (see also Kieren et al., 2018), although several studies deal with the reproductive ecology of *Echinotriton* (Utsunomiya et al., 1978; Xie et al., 2000; Sparreboom et al., 2001; Utsunomiya and Matsui, 2002; Igawa et al., 2013). Only little information is available for *Tylototriton*, often only from anecdotal observations (e.g. Gong and Mu, 2008; Phimmachak et al., 2015b). Although the mating mode and habitat of *E. maxiquadratus* are unknown, we could infer from phylogeny that its mating is terrestrial. Although various crocodile newt species are kept as pets for quite a long time, the origin and species affiliation of captive newts is often uncertain (Mudrack, 1972; Fleck, 2010a; 2010b). Various observations of the mating mode or habitat in *T. (T.) verrucosus* in captivity are available (Rehberg, 1986; Sparreboom, 1999; Jungnickel, 2007), but there is only circumstantial evidence that these observations are all attributable to currently described *T. (T.) verrucosus* or to other morphologically similar species. From published figures, it might be assumed that some observations rather deal with *T. (T.) shanjing* than *T. (T.) verrucosus* (Rehberg, 1986) leading to confusion. Whereas we report terrestrial mating for *T. (Y.) wenxianensis* based on Gong and Mu (2008) and Pasmans et al. (2017), Kieren et al. (2018) list aquatic mating for the species without any source of information. However, Gong and Mu (2008) observed clutch deposition under water, contrary to Pasmans et al. (2017). More studies on the ecology of crocodile newts are urgently needed to understand how reproductive strategies vary interspecifically and even between populations.

Despite the fragmentariness of data, some ecological signals are visible. A correlation between morphology and ecology is obvious for oviposition site, as *Echinotriton* and *Yaotriton* deposit their clutches on land, whereas members of *Tylototriton* subgenera deposit them in water bodies. For *T. (T.) podichthys,* terrestrial clutch deposition has been observed (Phimmachak et al., 2015b). Nevertheless, it is not apparent whether in this case cranial shape similarity among *Echinotriton* and *Yaotriton* is due to ecology or is constrained by phylogeny. In the latter case, *Tylototriton* subgenera would have re‐evolved aquatic oviposition, as *Pleurodeles* as the stem group also deposits clutches in water (Figure 3). Only the mating mode is correlated with cranial shape, simultaneously accounting for phylogeny, the different character states being randomly distributed within *Tylototriton.* How the different shapes might contribute to the different modes is yet speculative. In lateral view, amplectant species show a flatter shaped cranium. In dorsal view, those species exhibit a narrower head shape. This shape might be more streamlined, leading to lower resistance during swimming or crawling.

No cranial adaptations towards specific climate zones were detectable among extant crocodile newts and fossil *Chelotriton,* although the climate was markedly different when the deposits of Enspel and Randeck were formed. The mean annual temperature (MAT) was higher in central Europe than nowadays (Böhme, 2003; Uhl and Herrmann, 2010). The Randeck Maar dates back to the Mid‐Miocene climatic optimum, with a MAT of about 24°C (Böhme, 2003), whereas Enspel is about 10 Ma older than Randeck, with a MAT of between 15 and 17°C (Uhl and Herrmann, 2010), similar to the Mediterranean nowadays.

*Chelotriton* as a whole rather represents a subtropical to tropical distributed genus (Böhme, 2003; Uhl and Herrmann, 2010) as are the known distribution ranges of most extant crocodile newts. The relative long timespan between the two deposits investigated here, different climates and morphological disparity support the idea that different species were involved herein and in other European deposits. Westphal (1980) noticed that *Chelotriton* is rarely found in lake deposits and argued that *Chelotriton* was more terrestrial than other urodeles such as *Tylototriton*. This would coincide with our findings that Enspel‐*Chelotriton* more closely resembles *Echinotriton* and *Yaotriton*, which mate and lay their clutches terrestrially and spend less time in aquatic habitats. A more extended study including more material of *Chelotriton* from other deposits would be helpful to resolve intergeneric relationships of fossil in relation to extant newt species.

## 5 CONCLUSIONS

The different datasets (external morphology and osteology) were mostly congruent in their results, osteological datasets leading to a better separation of taxonomic units such as in *Pleurodeles*. With skulls, there are more possibilities to place accurate landmarks along bones and their sutures and the repeatability is higher compared with landmarks placed on soft tissues.

Cranial morphology of crocodile newts provides a congruent phylogenetic signal separating the subgenera. As we had no access to specimens of *T. pseudoverrucosus,* we could not draw a conclusion about the morphological distinctness and validity of *Liangshantriton* and rather follow the opinion placing *T. taliangensis* in *Tylototriton* subgenera. Within subgenera, phylogeny plays a minor role in the evolution of cranial shape in crocodile newts. Among reproductive traits, oviposition site was evidently correlated to phylogeny. This also supports the morphological similarity of *Echinotriton* and subgenus *Yaotriton,* both of which deposit clutches on land. It is not apparent in this case whether cranial shape represents an adaptation to ecology or rather is constrained by phylogeny. Mating mode was the only trait associated with cranial shape, simultaneously correcting for phylogeny. Climate zone had no effect on cranial shape of pleurodeline newts, confirming their quite conservative morphology (Hernandez et al., 2018). Fossil remains were partly distorted and not fully preserved, so that retrodeformation was applied and some landmarks were virtually reconstructed. Nevertheless, the analysis of data with and without fossils revealed a similar amount of morphological variation, promoting cranial shape conservatism in crocodile newts. But fossil *Chelotriton* showed a larger disparity in cranial shape and size in comparison with extant species, underpinning that *Chelotriton* represents a separate lineage of pleurodeline newts rather than a grade towards extant species groups (Schoch et al., 2015). *Chelotriton* from the deposits of Enspel and Randeck probably represent different species and cannot be assigned to *C. paradoxus* simultaneously. As skull morphology of fossil *Chelotriton* from Enspel closely resembles that of *Echinotriton* and *Yaotriton,* we conclude a more terrestrial ecology of the fossil pleurodeline newt. Further studies on the ecology of crocodile newts are urgently needed for two reasons: to better understand how ecology affects evolution of morphology and for conservation purposes, as crocodile newts are highly threatened by various factors driving them close to extinction in the near future (Rowley et al., 2010; et al.2016).

## AUTHOR CONTRIBUTIONS

P.P. – study design, specimen loans, CT‐scanning, data collection, data analysis, data interpretation, drafting manuscript. M.Z. – CT‐scanning, data collection. T.B. – CT‐scanning. R.R.S. – study design, specimen loans. A.K. – study design, specimen loans, data interpretation, drafting manuscript. All authors edited and approved the manuscript draft. The data that support the findings of this study are available from the corresponding author upon reasonable request.

## Supporting information

Fig S1

Fig S2

Fig S3

Table S1

Supplementary Material
