# Supplementary figures and images for: Cranial shape evolution of extant and fossil crocodile newts and its relation to reproduction and ecology
Source: J Anat. 2020 Apr 15;237(2):285–300. doi: 10.1111/joa.13201 (PMC7369190; doi:10.1111/joa.13201)

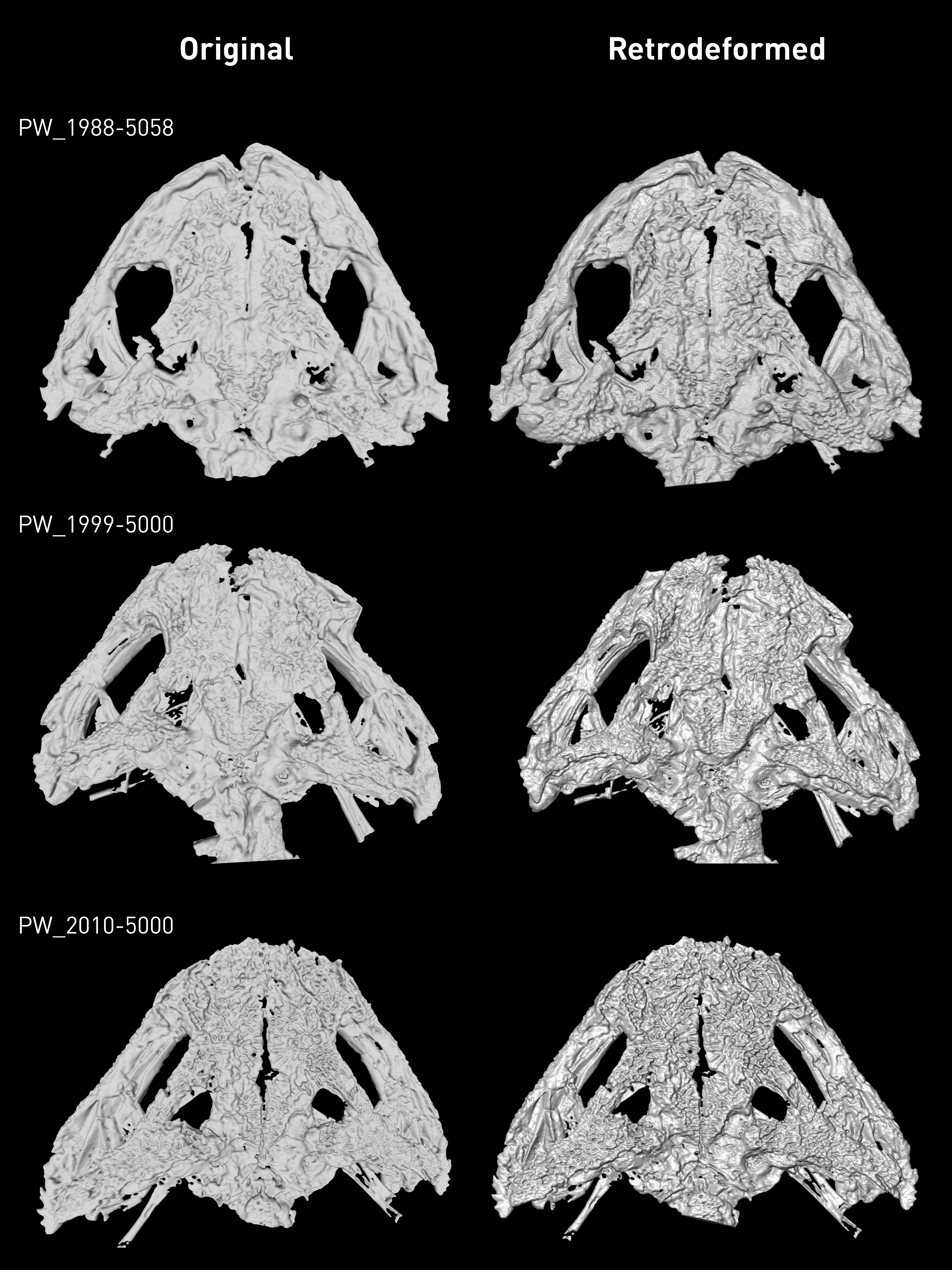

Supplement: Supplementary file 1 — Fig S1 [file JOA-237-285-s001.tif]

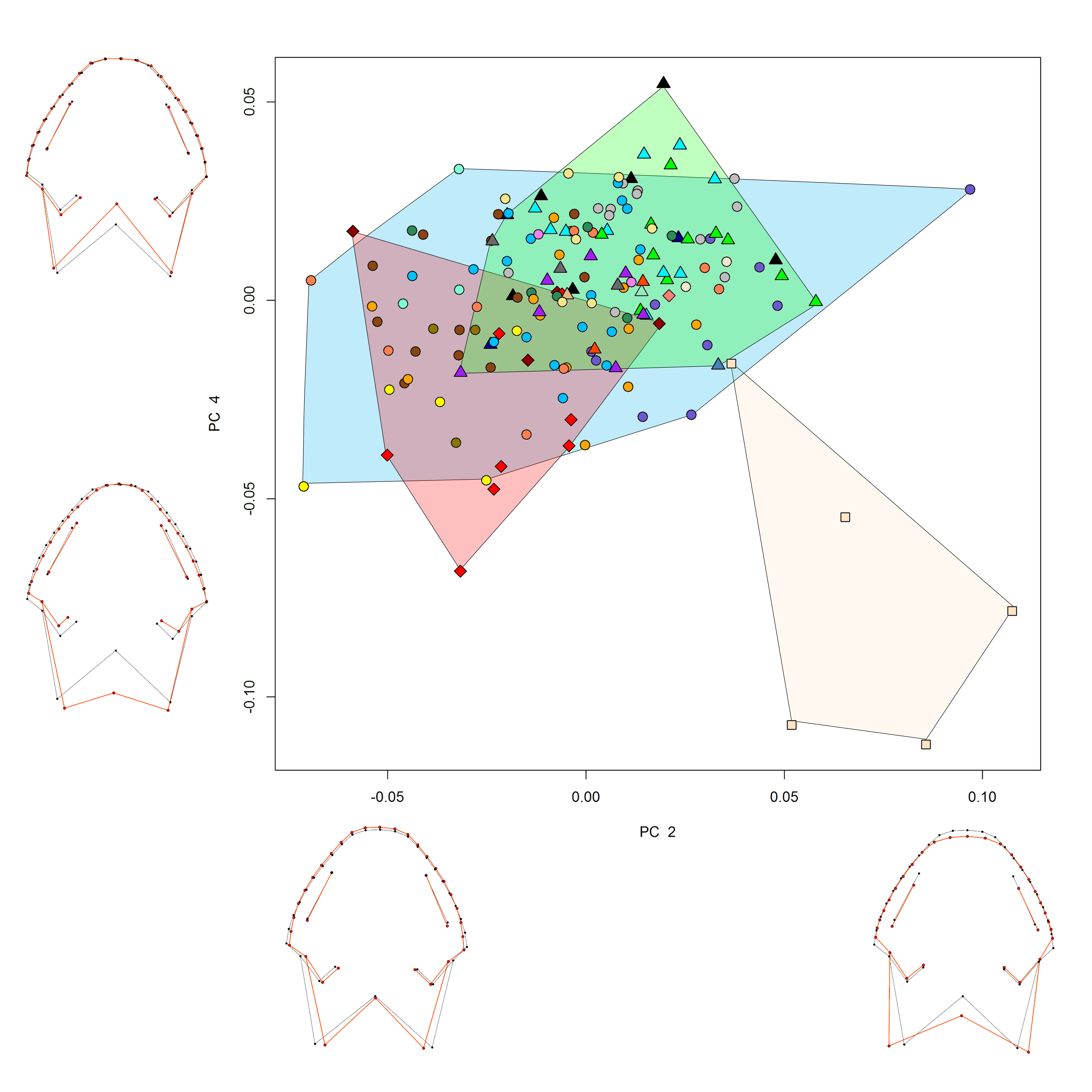

Supplement: Supplementary file 2 — Fig S2 [file JOA-237-285-s002.TIF]

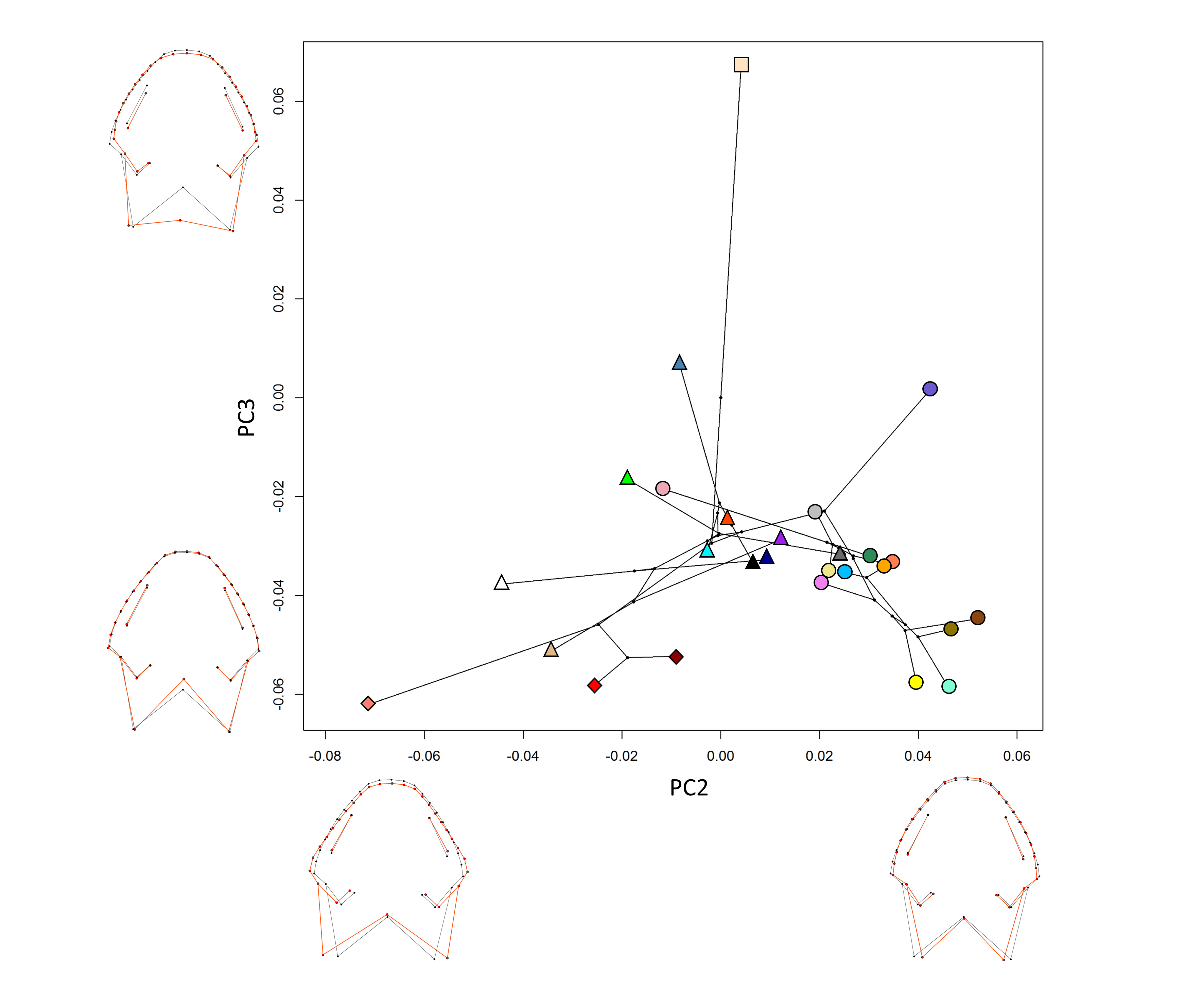

Supplement: Supplementary file 3 — Fig S3 [file JOA-237-285-s003.tif]
