# Supplementary material for: Cranial shape evolution of extant and fossil crocodile newts and its relation to reproduction and ecology
Source: J Anat. 2020 Apr 15;237(2):285–300. doi: 10.1111/joa.13201 (PMC7369190; doi:10.1111/joa.13201)
Supplement: Supplementary file 4 — Table S1 [file JOA-237-285-s004.docx]

# Table S1: Specimens of pleurodelin salamanders examined for cranial morphometrics analyses in external and osteological morphology. Holo- and paratype catalogue numbers are given in bold.

| Species | Catalogue no. | External morphology | Osteology | Comment |
| --- | --- | --- | --- | --- |
| Echinotriton andersoni - Okinawa | CAS22130 | X |  |  |
| *Echinotriton andersoni -* Okinawa | CAS22148 | X |  |  |
| *Echinotriton andersoni -* Okinawa | CAS22153 | X |  |  |
| *Echinotriton andersoni -* Okinawa | CAS22154 | X | X |  |
| *Echinotriton andersoni -* Okinawa | CAS22185 | X |  |  |
| *Echinotriton andersoni -* Okinawa | CAS22266 | X | X |  |
| *Echinotriton andersoni -* Okinawa | CAS22269 |  | X |  |
| *Echinotriton andersoni -* Okinawa | CAS22286 | X | X |  |
| *Echinotriton andersoni -* Okinawa | CAS22301 | X | X |  |
| *Echinotriton andersoni -* Amami | SMF70901 | X |  |  |
| *Echinotriton andersoni -* Amami | SMF86880 | X | X |  |
| *Echinotriton andersoni -* Amami | SMF86881 | X | X |  |
| *Echinotriton andersoni -* Amami | SMF86882 | X | X |  |
| *Echinotriton maxiquadratus* | **SY20131101ENT** | X |  | from Literature |
| *Tylototriton anguliceps* | **HNUE A.I.110** | X |  | from Literature |
| *Tylototriton anguliceps* | **HNUE A.I.1.109** | X |  | from Literature |
| *Tylototriton anguliceps* | NCSM82952 | X | X |  |
| *Tylototriton anguliceps* | NCSM82953 | X | X |  |
| *Tylototriton anguliceps* | **TBU PAE.671** | X |  | from Literature |
| *Tylototriton asperrimus* | SMNS14610 | X | X |  |
| *Tylototriton asperrimus* | ZFMK82728 | X | X |  |
| *Tylototriton asperrimus* | ZFMK82730 | X | X |  |
| *Tylototriton asperrimus* | ZFMK82735 | X | X |  |
| *Tylototriton asperrimus* | ZFMK85178 | X | X |  |
| *Tylototriton asperrimus* | ZFMK85179 | X | X |  |
| *Tylototriton asperrimus* | **ZMB34090** |  | X |  |
| *Tylototriton broadoridgus* | **HNUL840513527** | X |  | from Literature |
| *Tylototriton hainanensis* | NCSM78989 | X | X |  |
| *Tylototriton himalayanus* | MNHN1976.623 |  | X |  |
| *Tylototriton himalayanus* | MNHN1976.626 | X | X |  |
| *Tylototriton himalayanus* | MNHN1976.643 | X |  |  |
| *Tylototriton himalayanus* | MNHN1976.651 | X |  |  |
| *Tylototriton himalayanus* | MNHN1976.669 |  | X |  |
| *Tylototriton himalayanus* | MNHN1976.670 | X | X |  |
| *Tylototriton himalayanus* | MNHN1976.678 | X | X |  |
| *Tylototriton himalayanus* | MNHN1976.685 | X |  |  |
| *Tylototriton himalayanus* | MNHN1976.688 | X |  |  |
| *Tylototriton himalayanus* | MNHN1976.689 | X | X |  |
| *Tylototriton himalayanus* | MNHN1976.690 | X |  |  |
| *Tylototriton himalayanus* | MNHN1976.692 | X | X |  |
| *Tylototriton himalayanus* | MNHN1976.693 |  | X |  |
| *Tylototriton himalayanus* | SMF1135 | X | X |  |
| *Tylototriton himalayanus* | ZMB10026 | X |  |  |
| *Tylototriton kachinorum* | **ZDUM0101** | X |  | from Literature |
| *Tylototriton kachinorum* | **ZDUM0102** | X |  | from Literature |
| *Tylototriton kachinorum* | **ZDUM0103** | X |  | from Literature |
| *Tylototriton kachinorum* | **ZDUM0104** | X |  | from Literature |
| *Tylototriton kachinorum* | **ZDUM0105** | X |  | from Literature |
| *Tylototriton kachinorum* | **ZMMUA5953** | X |  | from Literature |
| *Tylototriton kachinorum* | **ZMMUA5954** | X |  | from Literature |
| *Tylototriton kweichowensis* | MTKD30364 | X |  |  |
| *Tylototriton kweichowensis* | MTKD30369 | X |  |  |
| *Tylototriton kweichowensis* | MTKD30372 | X |  |  |
| *Tylototriton kweichowensis* | MTKD31097 | X |  |  |
| *Tylototriton kweichowensis* | MTKD38093 | X |  |  |
| *Tylototriton kweichowensis* | USNM95518 | X | X |  |
| *Tylototriton kweichowensis* | USNM95520 | X | X |  |
| *Tylototriton kweichowensis* | USNM95524 | X | X |  |
| *Tylototriton kweichowensis* | USNM95562 | X | X |  |
| *Tylototriton kweichowensis* | USNM95563 | X | X |  |
| *Tylototriton liuyangensis* | **HNUL11053108** | X |  | from Literature |
| *Tylototriton lizhenchangi* | MTKD47802 | X | X |  |
| *Tylototriton lizhenchangi* | MTKD47859 | X | X |  |
| *Tylototriton lizhenchangi* | MTKD48158 | X |  |  |
| *Tylototriton ngarsuensis* | **LUSHC13763** | X |  | from Literature |
| *Tylototriton ngarsuensis* | **LUSHC13764** | X |  | from Literature |
| *Tylototriton notialis* | **FMNH271121** | X |  | from Literature |
| *Tylototriton notialis* | NCSM80315 | X | X |  |
| *Tylototriton panhai* | NCSM82954 | X | X |  |
| *Tylototriton panhai* | NCSM82955 | X | X |  |
| *Tylototriton panhai* | NCSM82956 | X | X |  |
| *Tylototriton panwaensis* | CAS245290 | X | X |  |
| *Tylototriton panwaensis* | **CAS245418** | X | X |  |
| *Tylototriton panwaensis* | **CAS245426** | X | X |  |
| *Tylototriton podichthys* | NCSM86520 | X | X |  |
| *Tylototriton podichthys* | ZFMK95521 | X | X |  |
| *Tylototriton podichthys* | ZFMK95522 | X | X |  |
| *Tylototriton shanorum* | **CAS230940** | X |  | from Literature |
| *Tylototriton shanorum* | ZFMK83199 | X | X |  |
| *Tylototriton shanorum* | ZFMK83200 | X |  |  |
| *Tylototriton shanorum* | ZFMK83202 | X | X |  |
| *Tylototriton shanorum* | ZFMK83203 | X | X |  |
| *Tylototriton shanorum* | ZFMK83204 | X | X |  |
| *Tylototriton shanjing* | CAS215118 | X | X |  |
| *Tylototriton shanjing* | CAS215119 | X | X |  |
| *Tylototriton shanjing* | CAS215120 | X | X |  |
| *Tylototriton shanjing* | CAS242484 | X |  |  |
| *Tylototriton shanjing* | CAS242518 | X |  |  |
| *Tylototriton shanjing* | CAS242535 | X | X |  |
| *Tylototriton shanjing* | ZFMK83208 | X | X |  |
| *Tylototriton shanjing* | ZFMK83209 | X |  |  |
| *Tylototriton shanjing* | ZMB73596 | X | X |  |
| *Tylototriton shanjing* | ZMB73597 | X | X |  |
| *Tylototriton shanjing* | ZMB73598 | X | X |  |
| *Tylototriton shanjing* | ZMB73599 | X |  |  |
| *Tylototriton shanjing* | ZMB73600 | X | X |  |
| *Tylototriton taliangensis* | MTKD36799 | X |  |  |
| *Tylototriton taliangensis* | MTKD37682 | X | X |  |
| *Tylototriton taliangensis* | MTKD37790 |  | X |  |
| *Tylototriton taliangensis* | MTKD38088 |  | X |  |
| *Tylototriton taliangensis* | NHMW39889.12 | X | X |  |
| *Tylototriton taliangensis* | NHMW39889.16 | X | X |  |
| *Tylototriton taliangensis* | NHMW39889.18 | X | X |  |
| *Tylototriton taliangensis* | SMNS14609 | X |  |  |
| *Tylototriton taliangensis* | ZFMK83110 | X | X |  |
| *Tylototriton taliangensis* | ZFMK93752 | X | X |  |
| *Tylototriton taliangensis* | ZFMK93760 | X | X |  |
| *Tylototriton taliangensis* | ZFMK93762 | X | X |  |
| *Tylototriton uyenoi* | **KUHE19147** | X |  | from Literature |
| *Tylototriton uyenoi* | MNHN1987.3725 | X | X |  |
| *Tylototriton uyenoi* | MNHN1987.3731 | X | X |  |
| *Tylototriton uyenoi* | MNHN1987.3734 | X | X |  |
| *Tylototriton uyenoi* | MNHN1987.3739 | X |  |  |
| *Tylototriton uyenoi* | MNHN1987.3742 |  | X |  |
| *Tylototriton uyenoi* | MNHN1987.3749 | X |  |  |
| *Tylototriton uyenoi* | MNHN1987.3750 | X | X |  |
| *Tylototriton uyenoi* | MNHN1987.3752 | X |  |  |
| *Tylototriton uyenoi* | MNHN1987.3758 |  | X |  |
| *Tylototriton uyenoi* | MNHN1987.3759 | X |  |  |
| *Tylototriton uyenoi* | MNHN1987.3761 | X |  |  |
| *Tylototriton uyenoi* | MNHN1987.3765 | X | X |  |
| *Tylototriton uyenoi* | MNHN1987.3768 | X | X |  |
| *Tylototriton uyenoi* | SMNS15132 | X | X |  |
| *Tylototriton verrucosus* | CAS215065 | X | X |  |
| *Tylototriton verrucosus* | CAS215068 | X | X |  |
| *Tylototriton verrucosus* | CAS215071 | X | X |  |
| *Tylototriton verrucosus* | CAS215072 | X | X |  |
| *Tylototriton verrucosus* | CAS215075 | X | X |  |
| *Tylototriton verrucosus* | CAS215077 | X | X |  |
| *Tylototriton verrucosus* | CAS234480 | X | X |  |
| *Tylototriton verrucosus* | CAS245445 | X | X |  |
| *Tylototriton verrucosus* | CAS245449 | X |  |  |
| *Tylototriton verrucosus* | KIZ20130605? | X |  | from Literature |
| *Tylototriton verrucosus* | MNHN1887.223 | X | X |  |
| *Tylototriton verrucosus* | MNHN1893.529 | X | X |  |
| *Tylototriton verrucosus* | MNHN1893.530 | X |  |  |
| *Tylototriton verrucosus* | NHMW8608.1 | X | X |  |
| *Tylototriton verrucosus* | NHMW8608.2 | X | X |  |
| *Tylototriton verrucosus* | SMF1134 | X | X |  |
| *Tylototriton verrucosus* | SMNS1598.1 | X | X |  |
| *Tylototriton vietnamensis* | NHMW8607.1 | X |  |  |
| *Tylototriton vietnamensis* | NHMW8607.2 | X | X |  |
| *Tylototriton vietnamensis* | SMF1200 | X |  |  |
| *Tylototriton vietnamensis* | SMF83417 | X | X |  |
| *Tylototriton vietnamensis* | SMF83418 | X | X |  |
| *Tylototriton vietnamensis* | SMF83419 | X | X |  |
| *Tylototriton vietnamensis* | SMF83420 | X | X |  |
| *Tylototriton vietnamensis* | SMF83421 | X | X |  |
| *Tylototriton vietnamensis* | ZFMK86339 | X | X |  |
| *Tylototriton vietnamensis* | ZFMK92049 | X |  |  |
| *Tylototriton vietnamensis* | ZFMK95519 | X | X |  |
| *Tylototriton wenxianensis* | SMNS15012 | X | X |  |
| *Tylototriton wenxianensis* | SMNS15013 | X | X |  |
| *Tylototriton wenxianensis* | ZFMK83738 | X |  |  |
| *Tylototriton wenxianensis* | ZFMK83739 | X | X |  |
| *Tylototriton wenxianensis* | ZFMK83740 | X | X |  |
| *Tylototriton wenxianensis* | ZFMK83741 | X | X |  |
| *Tylototriton wenxianensis* | ZFMK83744 | X |  |  |
| *Tylototriton yangi* | no voucher | X |  | Live specimen |
| *Tylototriton yangi* | no voucher | X |  | Live specimen |
| *Tylototriton ziegleri* | ROM35327 | X | X |  |
| *Tylototriton ziegleri* | ROM35328 | X |  |  |
| *Tylototriton ziegleri* | ROM35330 | X | X |  |
| *Tylototriton ziegleri* | ROM35333 | X |  |  |
| *Tylototriton ziegleri* | ROM35334 | X | X |  |
| *Tylototriton ziegleri* | ROM35338 | X |  |  |
| *Tylototriton ziegleri* | ROM35339 | X | X |  |
| *Tylototriton ziegleri* | ROM35342 | X |  |  |
| *Tylototriton ziegleri* | ROM35343 |  | X |  |
| *Tylototriton ziegleri* | ROM35344 | X |  |  |
| *Tylototriton ziegleri* | ROM35346 | X |  |  |
| *Pleurodeles waltl* | SMNS13466 | X | X |  |
| *Pleurodeles waltl* | SMNS1563 | X | X |  |
| *Pleurodeles waltl* | SMNS1564 | X | X |  |
| *Pleurodeles waltl* | ZSM5505-2005 | X | X |  |
| *Pleurodeles waltl* | ZSM5506-2005 | X | X |  |
| *Chelotriton -* Enspel | PW_1998-5058 |  | X |  |
| *Chelotriton -* Enspel | PW_1999-5000 |  | X |  |
| *Chelotriton -* Enspel | PW_2001-5002 |  | X |  |
| *Chelotriton -* Enspel | PW_2001-5010 |  | X |  |
| *Chelotriton -* Enspel | PW_2006-5031A |  | X |  |
| *Chelotriton -* Enspel | PW_2010-5000 |  | X |  |
| *Chelotriton -* Enspel | PW_2015-5163 |  | X |  |
| *Chelotriton -* Randeck | SMNS80672 |  | X |  |

# Table S2: 2D landmark definitions on external and osteological cranial images for geometric morphometrics analyses in pleurodeline newts.

| Head Dorsal View | |
| --- | --- |
| 1 | Tip of snout |
| 2-3 | Medial canthus |
| 4-5 | Lateral canthus |
| 6-7 | Widest point of maxillaries |
| 8-9 | Distal point of dorso-lateral ridges |
| 10-11 | Posterior point of dorso-lateral ridges |
| 12-13 | Proximal point of dorso-lateral ridge in the posterior part |
| 14-15 | Posterior point of parotoids |
| 16 | Anterior point of vertebral ridge |
| 17-36 | Semilandmarks along snout |
|  |  |
| Head Lateral View | |
| 1 | Anterior naris edge |
| 2 | Anterior point of oral fissure |
| 3 | Posterior point of oral fissure |
| 4 | Medial canthus |
| 5 | Lateral canthus |
| 6 | Ventral edge of eye |
| 7 | Posterior point of dorso-lateral ridge |
| 8-27 | Semilandmarks along cranial roof |
|  |  |
| Skull Dorsal View | |
| 1 | Suture among premaxilla |
| 2 | Anterior suture of frontals |
| 3 | Posterior suture of frontals |
| 4 | Posterior suture of parietals |
| 5-6 | Tip of occipital condyle |
| 7-8 | Distal point of suture among frontals and parietal |
| 9-10 | Tip of pterygoid |
| 11-12 | Proximal tip of dorsolateral ridge at posterior part |
| 13-14 | Posterior end of squamosal |
| 15-16 | Distal point of processus alaris frontalis |
| 17-18 | Posterior point of prefrontal |
| 19-20 | Anterior orbit edge |
| 21-22 | Posterior point of maxilla (connection with quadratum) |
| 23-42 | Semilandmarks along maxilla and premaxilla |
| 43-62 | Semilandmarks along distal edge of squamosal |
| 63-72 | Semilandmarks along distal edge of frontal |
| 73-82 | Semilandmarks along distal edge of prefrontal |
|  |  |
| Skull Lateral view | |
| 1 | Posterior point of occipital condyle |
| 2 | Posterior point of squamosal |
| 3 | Posterior point of quadratojugale |
| 4 | Tip of maxillary |
| 5 | Dorsal point of maxillary in posterior half |
| 6 | Ventral point of prefrontal |
| 7 | Suture among maxillary and nasale |
| 8 | Suture among maxillary and premaxillary at naris |
| 9 | Suture among maxillary and premaxillary at tooth row |
| 10 | Anterior point of premaxillary at tooth row |
| 11-30 | Semilandmarks along cranial roof |
| 31-40 | Semilandmarks along tooth bearing edge of maxillary |
